# BLM helicase protein negatively regulates stress granule formation through unwinding RNA G-quadruplex structures

**DOI:** 10.1093/nar/gkad613

**Published:** 2023-07-28

**Authors:** Yehuda M Danino, Lena Molitor, Tamar Rosenbaum-Cohen, Sebastian Kaiser, Yahel Cohen, Ziv Porat, Hagai Marmor-Kollet, Corine Katina, Alon Savidor, Ron Rotkopf, Eyal Ben-Isaac, Ofra Golani, Yishai Levin, David Monchaud, Ian D Hickson, Eran Hornstein

**Affiliations:** Department of Molecular Genetics, Weizmann Institute of Science, Rehovot 7610001, Israel; Department of Molecular Neuroscience, Weizmann Institute of Science, Rehovot 7610001, Israel; Department of Molecular Genetics, Weizmann Institute of Science, Rehovot 7610001, Israel; Department of Molecular Neuroscience, Weizmann Institute of Science, Rehovot 7610001, Israel; Department of Molecular Neuroscience, Weizmann Institute of Science, Rehovot 7610001, Israel; Department of Brain science, Weizmann Institute of Science, Rehovot 7610001, Israel; Center for Chromosome Stability, Dept. of Cellular and Molecular Medicine, Panum Institute, Copenhagen Univ, 2200 København N., Denmark; Department of Molecular Genetics, Weizmann Institute of Science, Rehovot 7610001, Israel; Department of Molecular Neuroscience, Weizmann Institute of Science, Rehovot 7610001, Israel; Flow Cytometry Unit, Life Sciences Core Facilities, Weizmann Institute of Science, Rehovot 7610001, Israel; Department of Molecular Genetics, Weizmann Institute of Science, Rehovot 7610001, Israel; 1E therapeutics, Rehovot, Israel; de Botton Institute for Protein Profiling, The Nancy and Stephen Grand Israel National Center for Personalized Medicine, Weizmann Institute of Science, Rehovot 7610001, Israel; de Botton Institute for Protein Profiling, The Nancy and Stephen Grand Israel National Center for Personalized Medicine, Weizmann Institute of Science, Rehovot 7610001, Israel; Bioinformatics Unit, Life Sciences Core Facilities, Weizmann Institute of Science, Rehovot 7610001, Israel; MICC Cell Observatory Unit, Life Sciences Core Facilities, Weizmann Institute of Science, Rehovot 7610001, Israel; MICC Cell Observatory Unit, Life Sciences Core Facilities, Weizmann Institute of Science, Rehovot 7610001, Israel; de Botton Institute for Protein Profiling, The Nancy and Stephen Grand Israel National Center for Personalized Medicine, Weizmann Institute of Science, Rehovot 7610001, Israel; Institut de Chimie Moleculaire, ICMUB CNRS UMR 6302, uB Dijon, France; Center for Chromosome Stability, Dept. of Cellular and Molecular Medicine, Panum Institute, Copenhagen Univ, 2200 København N., Denmark; Department of Molecular Genetics, Weizmann Institute of Science, Rehovot 7610001, Israel; Department of Molecular Neuroscience, Weizmann Institute of Science, Rehovot 7610001, Israel

## Abstract

Bloom's syndrome (BLM) protein is a known nuclear helicase that is able to unwind DNA secondary structures such as G-quadruplexes (G4s). However, its role in the regulation of cytoplasmic processes that involve RNA G-quadruplexes (rG4s) has not been previously studied. Here, we demonstrate that BLM is recruited to stress granules (SGs), which are cytoplasmic biomolecular condensates composed of RNAs and RNA-binding proteins. BLM is enriched in SGs upon different stress conditions and in an rG4-dependent manner. Also, we show that BLM unwinds rG4s and acts as a negative regulator of SG formation. Altogether, our data expand the cellular activity of BLM and shed light on the function that helicases play in the dynamics of biomolecular condensates.

## Graphical Abstract

**Graphical Abstract:**
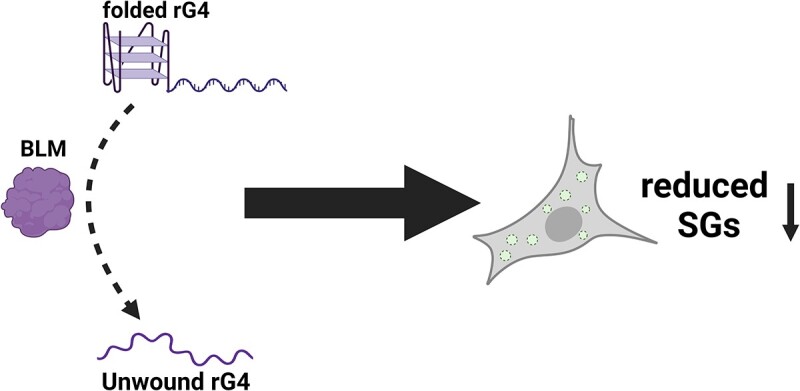

## INTRODUCTION

DNA or RNA G-quadruplexes (dG4s or rG4s) are nucleic acid secondary structures that are formed in guanine (G)-rich sequences (1–3). Thousands of such G4-forming sequences have been identified in the human genome (DNA) and transcriptome (RNA), on the basis of sequence predictions using bioinformatic tools and high-throughput sequencing-based methods (2,4–10). dG4s are highly polymorphic structures (parallel, anti-parallel, and hybrid G4s) that are involved in the control of replication, genome stability, and transcription (3). In contrast, rG4s primarily form parallel structures (1,11), and play regulatory roles in RNA splicing, mRNA stability, RNA transport, translation and stress response (1,6,11,12).

Complex biological processes involving G4s rely on the involvement of specific G4-binding proteins. The cellular localization of these proteins, their target specificity, binding affinity, and enzymatic activity contribute to defining their function. In this context, G4 helicases unwind dG4 and/or rG4 structures (13,14). Since stable dG4s could impair key biological processes, such as replication, transcription, translation and repair, these structural blocks must be unwound during these DNA transactions (11).

The biology of cytoplasmic rG4s is proposed to be linked, at least in part, to the cellular stress response (12) *via* the formation of stress granules (SGs) (6,15–17). SGs are cytoplasmic biomolecular condensates mainly composed of untranslated mRNAs and RNA-binding proteins (16,18–20). These condensates regulate RNA metabolism notably by halting mRNA translation (18,21–24).

The connection between SGs and rG4s is further substantiated by the fact that several rG4 binding or unwinding proteins have been found in SGs (6,15,25,26). Transfection of exogenous RNA with a preference to form rG4 promotes SG formation (15) and the rG4-helicase DHX36 remarkably affects SG formation (17). In addition, the SG core protein, RAS GTPase-activating binding protein 1 (G3BP1), is an rG4-binding protein and its interactions with rG4s have been suggested to contribute to SG formation (6,25,27). Together, these data imply that rG4s are key regulatory factors in SG biology.

Bloom's syndrome helicase (BLM) is one of the first human helicases reported to resolve dG4, requiring a 3′ single-stranded overhang for enzyme loading (28,29). The gene encoding the BLM protein is mutated in patients with Bloom's syndrome, displaying high genomic instability and predisposition to cancers (30,31). In addition, we have previously reported that BLM resides in SGs, under sodium arsenate stress (32). However, BLM’s regulatory role in the control of rG4s and in the biology of SGs has not yet been studied.

Here, we demonstrate that BLM is enriched in SGs under several stress conditions and also binds rG4s, thus expanding its reported functionality beyond dG4s. Moreover, we show that BLM is recruited to SGs in an rG4-dependent manner and negatively regulates their formation. These observations provide new insights into the cellular regulation of the stress response and more broadly into the functions of G4 helicases in biomolecular condensates.

## MATERIALS AND METHODS

### Reagents

MgCl2 (Mallinckrodt CHEMICALS, 6066-04), 100% Glycerol (Sigma-Aldrich, G5516), RNase/DNase-free UPW (Invitrogen, 10977-035), 1 M Tris–HCl pH 8.0 (Invitrogen, 15568–025), 1 M Tris–HCl pH 7.5 (Invitrogen, 15567-027), 0.5M EDTA (Invitrogen, AM9261), 1M DTT (Sigma-Aldrich, 43816), Tween-20 (Sigma-Aldrich, P1379), TBE ×10 (Fisher BioReagents, BP13334). Stock solutions of 4 M of NaCl (J.T. Baker, 0277), 3 M KCl (powder; MERCKGaA, 104936), 3 M LiCl (powder; J.T. Baker, 2370–01), 50mM ATP (Jena Bioscience, NU-1010) or 50 mM ATPgS (Jena Bioscience, NU-406) were prepared by dissolving the powders in RNase-free UPW or in UPW, treated with DEPC (Sigma-Aldrich, D5758) before use.

### RNA/DNA oligos

Chemically synthesized DNA or RNA oligonucleotides (6FAM/Dabcyl-labelled or unlabelled; Supplementary data 1, Datasheet S1) were from Sigma-Aldrich/MERCK or from Integrated DNA Technologies (IDT). We dissolved the oligonucleotides in RNase-free TEx1 buffer (10 mM Tris–HCl pH 7.5 and 1 mM EDTA) for the stock concentration of 100 uM and stored them at −80°C in aliquots to avoid thaw-freeze cycles.

### RNA/DNA G4 preparation

We diluted the FAM-labelled oligonucleotides to desired concentration in a TE 1× buffer with or without 150 mM DEPC-treated KCl or 150 mM DEPC-treated LiCl. Then, using a PCR machine RNAs folded to create secondary structures by heating to 90°C for 5 min and then lowering the temperature to 25°C in 5°C intervals (from 95°C to 50°C and from 30°C to 25°C) or in 10°C intervals (between 50°C to 30°C) as follows: 85 - 70°C for 5 min each, 65–50°C for 15 min each, 40–30°C for 30 min each and 25°C for 2 hr. All samples were stored at 4°C.

### Circular dichroism (CD) spectroscopy

We performed CD experiments at 25°C using Chirascan™-plus ACD spectropolarimeter with a quartz cuvette with a 1 mm path length. We collected CD spectra from 360 to 210 nm. The bandwidth was 1 nm, and the response time was 1 s. CD spectra signal corrected to background (buffer only) and represented the average of 3 runs.

### Cloning, expression and purification of recombinant core BLM protein

A truncated BLM 636–1298 (cBLM, spanning the helicase, RQC and HRDC domains) was expressed in E. coli and purified as described previously (33,34) with the addition of a MonoS ion-exchange- and gel-filtration step.

### Electro-mobility shift assays (EMSA)

We prepared 20 ul reaction mixtures, which contained 80 nM 5′-6FAM-labelled DNA or RNA oligonucleotides of G4-forming sequences (Supplementary data 1, Datasheet S1), and binding buffer consisting of 50 mM Tris–HCl pH 7.5, 50 mM NaCl, 1 mM DTT, 2 mM MgCl_2_ and 0.1% Tween-20. For RNA oligonucleotides we also added Ribolock (1:40; Thermo Scientific, E00381), with or without recombinant human core-BLM protein (50 or 150 nM). For the reactions with QUMA-1 (MERCK, SCT056), we added to the samples 1 uM QUMA-1. We incubated the binding reactions at 37°C for 30 min and then loaded them onto a 5% native non-denaturing polyacrylamide (acrylamide:bis-acrylamide 29:1 (30%); Bio-Lab, UN3426) gel consisting of (for 12.5 ml) 9 ml DEPC-DDW, 1.25 ml TBE 10×, 2.075 ml 30% polyacrylamide, 125 ul 10% Ammonium Persulfate (APS; Bio-Rad, 1610700) and 12.5 ul TEMED (Bio-Rad, 1610801). We performed electrophoresis at 70 V for 70 min in TBE 1× buffer on ice and in the dark. After 70 min, we performed gel scanning using ImageQuant LAS4000 (GE Healthcare) gel imager at cy2 channel (488 nm).

### Helicase activity *in vitro* assay

A mixture of 1 uM Dabcyl-labelled oligonucleotide (Dabcyl-S-rG4-VEGFA-U15) and 0.85 uM 6FAM-labelled oligonucleotide (F-rShort- 6FAM) was prepared in 20 mM Tris–HCl buffer (pH 7.5) containing 5 mM MgCl2, 1 mM KCl, 99 mM NaCl and RNase-free UPW. The 1.2-fold excess of the dabcyl-labelled strand ensured complete hybridization of the 6FAM-labelled strand and maximal quenching of the fluorescent signal. The mixture was annealed by PCR program as follows: 90°C, 5 min; 80°C, 10 min; 70°C, 10 min; 60°C, 1 h; 50°C, 1 h; 40°C, 1 h; 30°C, 1 h; 25°C, 2 h; 4°C to end. The annealed samples were then stored at -80°C in aliquots. A competitor oligonucleotide (Trap oligo, 1 uM) was heated at 90°C for 5 min and then cooled on ice for 15 min before the reaction. Oligonucleotides described in Supplementary Data 1, Datasheet S1). Helicase reactions were performed based on Mendoza *et al.* (35), with adaptations. The reactions were conducted in triplicates in 96-well plates (Nunc MicroWell 96-Well, black, Flat-Bottom Microplate, Thermo Fisher; Cat.# 137103) at 37°C with the lid. Fluorescence was monitored using a microplate reader (Tecan Infinite 200 PRO). Each replicate consisted of a 50 ul solution containing 40 nM 6FAM-Dabcyl system (pre-annealed), varying amounts of cBLM protein, and 200 nM Trap oligonucleotide (unlabelled and complementary to the FAM-labelled strand). Subsequently, 5 ul of a 50 mM ATP/ATPgS solution (5 mM final concentration/reaction) was added to each well. The 96-well plate was stirred for 10 s, and fluorescence emission was recorded every 15 s at excitation / emission wavelengths of 492 nm/525 nm, respectively.

### Cell culture

U2OS cell (Human Bone Osteosarcoma Epithelial Cells) and human Retinal Pigment Epithelial (RPE) cells were used, either BLM knockout (BLM KO) or wild-type (wt) (36). Cells were cultured in growth media consisting of Dulbecco's modified Eagle's medium (DMEM, Biological Industries, 01-050-1A) supplemented with 1% penicillin-streptomycin (Sartorius, 03-031-1B), and 10% fetal bovine serum (FBS, Sartorius, 04-007-1A), at 37°C, with 5% CO_2_. BJ fibroblasts were cultured in RPMI medium (Gibco, ThermoFisher Scientific, 21875–034) supplemented with 10% fetal bovine serum, at 37°C, with 5% CO_2_. For live imaging and staining experiments, we used G3BP1-GFP stably expressing U2OS cells. For proteomics and validation experiments for APEX proximity labelling, we used tetracycline-inducible G3BP1-APEX or NES-APEX expressing U2OS cells, as described previously (32). For SG-BLM colocalization studies, human iPSCs seeding on cover-slips, were done as described in Fernandopulle *et al.* (37). Finally, eight days old iPSC-derived neurons were treated with sodium arsenate (400 uM, 30 min), prior to fixation.

### siRNAs

siGenome siRNAs against Dhx36 (M-013167-00) and Blm (M-007287-02) genes, as well as siGENOME non-targeting siRNA #5 control (named here as ‘siControl’; D-001210–05) were from Dharmacon (Dharmacon siGENOME Human/Mouse/Rat SMARTpool). For transfection, we mixed siRNAs with Lipofectamine 2000 (Invitrogen; 11668019) and incubated them in Opti-MEM (Gibco; 11058021) for 20 min. Then, the cells were incubated with the solution in transfection media (DMEM and 10% FBS, without penicillin- streptomycin) for at least 4 h up to over-night (o.n) and afterward, we replaced the transfection media to complete medium for 48 h.

### APEX proximity labelling and pull-down

16 M Tetracycline-induced G3BP1-APEX or NES-APEX expressing U2OS cells were seeded per flask, in 180T flasks (90–100% confluency) with a medium which was supplemented with tetracycline for 24 h (50 ng/ml for each of the APEX baits) for inducing the APEX-bait gene expression. On the day of the experiment, the cells were incubated for 3 h with 1 uM QUMA-1, or an equivalent volume of DMSO prior to 150 uM Sodium arsenate (Sigma-Aldrich, 71287) stress for 2.5 h. Labelling activity was induced by supplementing Biotin-phenol (BP, 500 uM, Iris Biotech GmbH, LS-3500) for the last 30 min of the stress and then H_2_O_2_ 30% (1 mM, J.T.Baker 7722-84-1) was added for 1 min. APEX activity extinguished with quenching solution (sodium azide (10 mM, Mallinckrodt, 1953-57), sodium ascorbate (10 mM, Sigma-Aldrich, A7631) and Trolox (5 mM, Sigma-Aldrich, 238813) in PBS 1× twice for 1 min each time. Then, the cells were washed two more times with PBS 1×, 1 min each time, and for the last wash with quencher solution as mentioned before. After the fifth wash, the cells were scraped in quencher solution, centrifuged at 800 × g for 10 min at 4°C, pelleted and lysed in ice-cold RIPA lysis buffer supplemented with cOmplete Protease Inhibitor Cocktail (Roche, 4693116001) and PhosSTOP (Roche, 4906837001). Lysates were centrifuged at 14 000 × g for 10 min at 4°C. Protein concentration was quantified with Bio-Rad Protein Assay Dye Reagent (‘Bradford’; Bio-Rad, 500-0006). Streptavidin-coated magnetic beads (Pierce Streptavidin Magnetic Beads, Thermo-Fisher, 88816) were incubated for pulldown experiments with the ratio between extract: beads as 1 mg:200 ul, respectively (for proteomics we used 500 ug of the extract with 100 ul beads per sample, in a complete volume of 500 ul) with rotation overnight at 4°C. Then, the beads were washed twice with RIPA containing quencher solution, once with 1 M KCl solution, once with 0.1 M Na_2_CO_3_ solution (powder, Sigma-Aldrich, S7795), once with 2 M Urea solution (2 M urea; powder, Sigma-Aldrich, U0631, and 10 mM Tris-HCl pH 8.0) and for proteomics, each wash was done for 3 min and the biotinylated proteins were transferred to on-bead digestion process by trypsinization.

### Liquid chromatography and mass spectrometry

ULC/MS grade solvents were used for all chromatographic steps. Dry-digested samples were dissolved in 97:3% H_2_O/acetonitrile + 0.1% formic acid. Each sample was loaded and analyzed using split-less nano-Ultra Performance Liquid Chromatography (10 kpsi nanoAcquity; Waters, Milford, MA, USA). The mobile phase was: A) H2O + 0.1% formic acid and B) acetonitrile + 0.1% formic acid. Desalting of the samples was performed online using a Symmetry C18 reversed-phase trapping column (180 um internal diameter, 20 mm length, 5 um particle size; Waters). The peptides were then separated using a T3 HSS nano-column (75 um internal diameter, 250 mm length, 1.8 um particle size; Waters) at 0.35 ul/min. Peptides were eluted from the column into the mass spectrometer using the following gradient: 4 to 25%B in 155 min, 25 to 90%B in 5 min, maintained at 90% for 5 min and then back to initial conditions.

For MS, the nanoUPLC was coupled online through a nanoESI emitter (10 um tip; New Objective; Woburn, MA, USA) to a quadrupole orbitrap mass spectrometer (Q Exactive Plus, Thermo Scientific) using a FlexIon nanospray apparatus (Proxeon).

Data was acquired in data-dependent acquisition (DDA) mode, using a Top10 method. MS1 resolution was set to 70 000 (at 200 *m*/*z*), a mass range of 375–1650 *m*/*z*, AGC of 3e6, and maximum injection time was set to 60 ms. MS2 resolution was set to 17500, quadrupole isolation 1.7 *m*/*z*, AGC of 1e5, dynamic exclusion of 45 s, and maximum injection time of 60 msec.

### Raw proteomic data processing

The processing step was performed as described previously (32). In short, we processed the raw MS data by using MaxQuant version 1.6.2.6 (38), and a database search was done with the Andromeda search engine (39,40) by using the human Uniprot database. The data were filtered with a threshold of 1% the false discovery rate (FDR) for both the peptide-spectrum matches and the protein levels. The label-free quantification (LFQ) algorithm in MaxQuant (41) was utilized to compare experimental samples, except for the negative controls. Additional settings included variable modifications. The ‘match between runs’ option was enabled to transfer identification between separate LC–MS/MS runs based on their accurate mass and retention time after retention time alignment.

### Proteomic statistical analysis

ProteinGroups output table was imported from MaxQuant to Perseus environment version 1.6.2.3 (42) and analyzed with Perseus and then with R version 4.0.5 (43). We excluded reverse proteins, proteins identified only based on a modified peptide, and contaminants as quality control steps. Non-specific streptavidin-bead binding proteins were excluded by the following protocol: Intensity values were log_2_-transformed, and protein groups were filtered to retain only proteins with at least 2 valid values/group. Missing values were replaced by a constant low value (15). Student's *t*-test with S0 = 0.1 was performed with FDR *P*-value ≤0.05 and Fold Change (FC) >0, for pairs of APEX-On and corresponding APEX-Off samples for each group of condition/ treatment (DMSO/ NES-APEX; DMSO/ G3BP1-APEX; QUMA-1/ NES-APEX; QUMA-1/G3BP1-APEX). Proteins that passed all QC filters were separated for each condition (DMSO or QUMA-1). Within each condition, the LFQ intensities of G3BP1-APEX samples were compared to the LFQ intensities of NES-APEX samples to characterize the stress-granule associated proteins under each condition, as follows: Data were filtered to retain only proteins with at least two LFQ values in at least 1 group. Importantly, through the analysis, one repeat from the G3BP1-APEX samples under DMSO treatment (sample 1) was excluded because of suboptimal correlation with the other samples from this group. Missing data were imputed by creating an artificial normal distribution with a downshift of 1.5 standard deviations and a width of 0.4 of the original ratio distribution. Student's t-test called enriched SG proteins with S0 = 0.1 and FDR *P*-value ≤0.05 and a minimum of two-fold enrichment of proteins in G3BP1-APEX samples versus NES-APEX (log_2_(SG-APEX – NES-APEX) > 1). Following that, after filtered valid values of at least 20% in total, we imputed the LFQ intensities by creating an artificial normal distribution with a downshift of 1.8 standard deviations and a width of 0.3, and the G3BP1-APEX values per each condition (DMSO or QUMA-1) were normalized by the mean of their corresponding NES-APEX values. Then, we categorized the normalized G3BP1-APEX for two groups: DMSO and QUMA-1. We compared these two conditions by student's t-test with FDR correction by using R as mentioned above. Enrichment of proteins in SGs for DMSO or QUMA-1 treatment was determined by FDR p-value ≤ 0.05 and a minimum of two-fold enrichment (for DMSO: log_2_(QUMA-1 – DMSO) < –1; for QUMA-1: log_2_(QUMA-1 – DMSO) > 1). Principal component analysis (PCA), Volcano plot and Heatmap for comparison between DMSO and QUMA-1 SG proteomes were generated by Perseus and R.

### Mass spectrometry cBLM identification

Shifted G4 bands were cut from the gel and subjected to in-gel tryptic digestion followed by a desalting step. The resulting peptides were subjected to nanoflow liquid chromatography (nanoAcquity) coupled to high resolution, high mass accuracy mass spectrometry (Q Exactive HF, discovery mode). Raw data was processed using Proteome Discoverer version 2.4, and searched with SequestHT (44) and MS Amanda (45) against a protein reference list containing the recombinant cBLM sequence that we provided, the *E. coli* K12 protein database was downloaded from Uniprot.org and an in-house list of 128 common lab contaminants.

### Cell lysis and western blotting

For WB analysis of biotinylated proteins, the treatments and stress conditions as well as the APEX proximity labelling protocol were performed as described above. For BLM signal from pull-down of biotinylated proteins by APEX proximity labelling under DMSO or QUMA-1, we seeded 8–9 M tetracycline-inducible NES-APEX or G3BP1-APEX expressing U2OS cells with tetracycline in 80T flasks a day before the experiment. The wash steps were done except for the last steps, as follows: washes were done without prolonged incubation, and in addition to the wash steps above, the beads were washed twice more with RIPA buffer again and the biotinylated proteins were eluted from the beads by boiling (95°C) with 5× sample buffer supplemented with 2 mM free biotin for 10 min. Based on protein quantification with Protein Assay Dye Reagent, we loaded the beads 360 ug with 40 ul beads per sample, before the washing steps. The supernatant of each solution was taken for loading on the gel after the beads were magnetized.

For general pattern of biotinylated proteins by APEX proximity labelling under DMSO or QUMA-1, we seeded 500 K from the cell lines above in 6-well plates a day before the experiment. However, whole cell lysate was obtained by RIPA lysis buffer without pull-down and wash steps with Streptavidin beads. Based on protein quantification as mentioned, the protein was loaded 50 ug of total protein per well.

For WB analysis of BLM signal under siRNA treatment, we seeded 100 K G3BP1-GFP U2OS cells in 6-well plates and were transfected with siRNA control or siRNA against BLM in triplicates, as described above (see ‘siRNAs’ section). Then, we lysed the cells with RIPA lysis buffer, and based on protein quantification, the protein was loaded with 50 ug of total protein per well.

After lysis and preparation steps of the samples, for all the WB experiments, we resolved the proteins by 10% SDS–PAGE at 100 V for 10 min and then 120 V up to 80 min. After gel electrophoresis, proteins were transferred to nitrocellulose membranes (Whatman; 10401383) at 250 mA for 70 min. Membranes were blocked for 1 h at R.T. with 3% bovine albumin fraction V (MPBio; 160069) in PBS containing 0.05% Tween-20 (PBST) and for each experiment above, the procedure was done differently.

For WB analysis of BLM signal under siRNA treatment or in SGs after APEX proximity labelling and pull-down, we incubated the membranes with primary rabbit polyclonal antibody anti-BLM (1:500; abcam, ab476) overnight at 4°C with rocking in antibody solution (5% albumin, 0.02% sodium azide and five drops of phenol red in 0.05% PBST). Specifically for WB analysis of BLM signal under siRNA treatment, we used also incubated the membranes with primary monoclonal mouse antibody anti-Tubulin (1:2000; Sigma-Aldrich, T9026) as a control overnight at 4°C with rocking in antibody solution. Following primary antibodies incubation, membranes were washed three times for 5 min at R.T. with 0.05% PBST and were incubated for 1 hr at R.T. with horseradish peroxidase-conjugated species-specific secondary antibody. Specifically for WB analysis of biotinylated proteins, Streptavidin-HRP (1:1000; Sigma-Aldrich, Cat#RABHRP3) was used for 1 h at R.T in the dark.

For all the experiments we then washed the membranes three times for 5 min each in 0.05% PBST at R.T. and visualized them using EZ-ECL Chemiluminescence (Biological Industries, 20500-120) by ImageQuant LAS 4000 (GE Healthcare Life Sciences). Densitometric analysis was performed using Fiji software (NIH) and representative bands are presented.

### Staining and microscopy

50 K G3BP1-GFP expressing U2OS cells, BJ fibroblasts or iPSC-derived neurons seeded per well in 24-well plates on coverslips 24 hr prior to stress. After the stress induction, we fixed the cells with 4% PFA (Alfa Aesar, 43368) for 15 min at R.T. and washed them with RNase-free PBS 1X three times. Then, we treated the cells with 0.1% Triton-X for 15 min at R.T., blocked with CAS-Block reagent (ThermoFisher Scientific; 008120) for 10min at R.T., incubated with primary rabbit polyclonal anti-BLM antibody (1:100). Primary mouse monoclonal anti-G3BP1 antibody (1:200; Santa-cruz, sc-365338) incubated at cold room o.n. A day after, we washed the cells with RNase-free PBS three times, 5 min each, and then incubated with secondary Cy5-conjugated anti-Rabbit antibody (1:200) or also Cy2-conjugated anti-Mouse antibody (1:200, for non U2OS cells) for 1 h at R.T. Plates kept in the dark, washed with RNase-free PBS three times, 5 min each, dried and mounted with DAPI (Fluoroshield with DAPI; Sigma-Aldrich; F6057). SG induction, performed with NaAsO2 (400 uM for 30 min, Sigma-Aldrich, 71287) or Thapsigargin (1 uM for 1 h, Sigma-Aldrich, T9033), Puromycin (200 ug/ml for 4 h, Invivogen, ANT-PR) or by heat shock for 90 min at 43°C. A similar procedure was done for BLM staining in SGs as a result of DMSO versus QUMA-1 treatments (U2OS cells). Specifically for this experiment, the cells were incubated with DMSO or QUMA-1 (1 uM) for 3 hr prior to sodium arsenate stress (150 uM, 2.5 h).

For QUMA-1 staining in fixed cells, we seeded 12 K G3BP1-GFP expressing U2OS cells per well (in 96-well plates) 24 h prior to transfection, incubated the cells with 1 uM siRNAs (final concentration) against Dhx36, Blm, or siControl for 4-16 hr. 48 hr later, we fixed the cells with 4% PFA for 15 min and washed them with RNase-free PBS three times. Then, we incubated the cells with 2 uM QUMA-1 and Hoechst 33342 (1:8000; Sigma-Aldrich, B2261) for 10 min at 37°C. We kept the plate in the dark from this point. Next, we washed the cells with RNase-free PBS three times, 5 min each. We acquired the fixed cells without (BLM stainings) or with (QUMA-1 staining) taking z-stacks *via* a Zeiss LSM900 laser scanning confocal microscopy system equipped with a Zeiss Axiovert microscope and using a 63 × 1.4 NA oil immersion lens. Similar steps after fixation as well as image processing and analysis were done also for RPE cells (wt *versus* BLM KO).

For APEX proximity labelling validation in fixed cells, 50 K tetracycline-induced G3BP1-APEX or NES-APEX expressing U2OS cells were seeded 24 hr prior to stress. We incubated the cells with or without 400 uM sodium arsenate stress supplemented with or without 500 uM biotin-phenol for 30 min, and then the APEX proximity labelling was induced by the presence of H_2_O_2_ for 1 min. Next, the media was removed and the cells were washed three times with quencher solution (as mentioned above) and then fixed with 4% PFA for 15 min. After we washed them with PBS three times, the cells were treated with 0.1% Triton-X for 15 min at R.T., blocked with CAS-Block reagent for 10 min at R.T., and incubated with primary monoclonal anti-V5 tag (1:1000; ThermoFisher, R960-25), which represents the AEPX-bait proteins and goat polyclonal anti-TIA1 (1:50; Santa cruz, sc-1751) in a cold room o.n. A day after, we washed the cells with PBS three times, 5 min each, and then incubated them for 1 h at R.T. in the dark with secondary Cy5-conjugated anti-goat antibody, Cy2-conjugated anti-mouse antibody and NeutrAvidin-TexasRed conjugate (ThermoFisher, A2665) to stain the biotinylated proteins (for all 1:200). We washed the cells with RNase-free PBS three times, 5 min each, dried them and mounted them on slides with DAPI. We acquired the fixed cells *via* a Zeiss LSM800 laser scanning confocal microscopy system equipped with a Zeiss Axiovert microscope, and using a 63 × 1.4 NA oil immersion lens.

### Analysis of signal enrichment within stress granules

For analysis of the BLM enrichment signal, every particular enrichment value was analyzed per single SG and was determined as the signal-to-background ratio of the BLM intensity (cy5; purple) in the SG (G3BP1-GFP; green) compared to the fixed surrounded cytoplasmic area of the same SG in the U2OS cells. The analysis was done with Fiji software.

QUMA-1 enrichment signal, analyzed per single SG as the signal-to-background ratio of the rG4 (QUMA-1; red) intensity in the volume of the SG (G3BP1-GFP (U2OS cells) or cy2, anti-G3BP1 (RPE cells); green) compared to the fixed surrounded cytoplasmic volume of the same SG. The analysis was done with the Arivis software.

### Molecular cloning of mCherry-helicase overexpression

We cloned DHX36 isoform 1 CDS or BLM cDNA into mCherry-containing pUltraHot vector by using a restriction-free (RF) procedure with Q5 Hot start High-Fidelity DNA polymerase (NEB). The source of BLM cDNA was from a pTRIP-CMV-puro-2A-BLM plasmid (Addgene, plasmid #127641). The original plasmid for DHX36 CDS was a kind gift from Dr Daniel Benhalevy, Prof. Marcus Hafner and Prof. Katrin Paeschke.

### ImageStream analysis

Levels of QUMA-1 signal under knockdown of DHX36 and BLM. 800 K G3BP1-GFP expressing U2OS cells were seeded. A day after, the cells were transfected with siRNAs against DHX36, BLM, or siControl as described above (see ‘siRNAs’ section). Next, we incubated the cells with or without 150 uM sodium arsenate stress for 2.5 h. We fixed the cells with 4% PFA for 15 min at R.T. and washed them three times with PBS 1×, 5 min each time. Then, the cells were incubated with 0.5 uM QUMA-1 and Hoechst (1:8000) for 15 min at 37°C and were washed three times with PBS 1×, 5 min each. The cells were scraped and collected with PBS supplemented with 1% BSA. The cells were centrifuged gently (300 × g, 10 min at 4°C) and were suspended by quick vortex in the volume of 20–50 ul of PBS 1× with 1% BSA. Cells were imaged by an Imaging Flow Cytometer (ImageStreamX Mark II, AMNIS corp., Luminex, TX, USA). Data were acquired using a 40× lens, and the lasers used were 405 nm (20 mW), 488 nm (100 mW), 561 nm (20 mW) and 785 nm (1 mW). Data were analyzed using the manufacturer's image analysis software IDEAS 6.3 (AMNIS corp.). Images were compensated for spectral overlap using single-stained controls. Cells were selected for Hoechst positive cells by plotting the area of the DNA staining (AREA_M07, in square microns) *vs*. the intensity of the DNA staining (Intensity MC_Hoechst, arbitrary units). Cells positive for GFP expression were selected by plotting the intensity *vs*. Max Pixel (the value of the high-intensity pixel) of the GFP channel (ch02). To eliminate out-of-focus cells, cells were further gated using the Gradient RMS and contrast features (measures the sharpness quality of an image by detecting large changes of pixel values in the image). Single cells were selected by plotting again the area of Hoechst staining, *versus* the aspect ratio normalized for the intensity of the Hoechst staining (Aspect Ratio Intensity M07_Hoechst). Flat cells were further selected according to the intensity vs. Max Pixel values of the Hoechst staining. The normalized levels of QUMA-1 were calculated by dividing the total intensity (Intensity_MC_QUMA) by the cell area of the bright-field image (Area_M01).

For RPE cells (wt *versus* BLM KO), we performed the same fixation procedure as above, but we incubated the cells with 0.5 uM QUMA-1 3 hr prior to the fixation without any other treatment before fixation. After the fixation, we incubated the cells with Hoechst (1:8000) for 15 min at 37°C and were washed three times with PBS 1X, 5 min each. ImageStream analysis and the settings were the same as above.

Quantification of SGs in mCherry-positive cells. 400 K G3BP1-GFP expressing U2OS cells were seeded in 6-well plates. To generate mCherry overexpressing cells, 24 h later, the cells were transiently transfected with pUltraHot-mCherry, pUltraHot-mCherry-DHX36 or pUltraHot-mCherry-BLM expression plasmids, as described above. Stress induction, fixation, wash, and cell collection steps were performed as mentioned for the first experiment above (without QUMA staining but with Hoechst staining (1:800) to dye the nuclei). Quantification of SGs was taken into account only in mCherry overexpressing cells. The laser settings were the same as above, and mCherry was collected in channel 4. Cells positive for mCherry were selected by plotting the intensity *versus* Max Pixel of the mCherry channel. To identify cells with SGs, two truth populations were selected, and a classifier was created using the machine learning module in IDEAS 6.3. The percentage of cells with SGs within mCherry positive cells was quantified by plotting the granule classifier vs. the Max Pixel values of the GFP channel.

### Live-cell imaging

We seeded 12–15 K G3BP1-GFP expressing U2OS cells per well 24 or 48 h prior to the experiment in a 96 well plate (Brooks, MGB096-1-2-LG-L).

For experiments of SG formation under knock-down conditions, we transfected the G3BP1-GFP expressing U2OS cells with 1 uM siRNAs (final concentration) against Dhx36, Blm, or siControl, and incubated them for 4 h to on and then replaced the transfection medium to complete medium for 48 h.

For experiments of SG formation under overexpression conditions, we transfected the G3BP1-GFP expressing U2OS cells with pUltraHot-mCherry or pUltraHot-mCherry-DHX36 or with pUltraHot-mCherry-BLM by using jetOPTIMUS DNA transfection reagent (Polyplus; 101000051) and incubated them for 4 h and then replaced the transfection medium to complete medium. A day later, we induced overexpression with tetracycline.

48 hr after the transfection (both siRNAs or overexpression plasmids), we replaced the medium with a 150 uM NaAsO2-added medium and immediately took them to the microscope to monitor SG formation. We took SG live imaging by a PCO-Edge sCMOS camera controlled by VisView installed on a VisiScope Confocal Cell Explorer system (Yokogawa spinning disk scanning unit; CSU-W1) and an inverted Olympus microscope (60 × oil objective; excitation wavelength: GFP – 488 nm). We analyzed SG and cell areas using surface features in Imaris software 9.5.1.

### Real-time polymerase chain reaction (rt-PCR)

To validate the efficiency and the function of the siRNAs, we performed rtPCR on cDNA from G3BP1-GFP expressing U2OS cells after transfection with the siRNAs. Whole cell RNA extract was isolated from the cells by TRI reagent (Sigma-Aldrich, T9424) and RNA isolation kit (Direct-zol RNA miniprep; Zymo, R2051). Next, cDNA was generated from the extracted RNA by qScript cDNA synthesis kit (Quantabio, 95047). We performed a real-time PCR procedure using KAPA SYBR Fast qPCR kit Master Mix (2×) Prism ABI (Kapabiosystems, KK4604) and measured the amplification cycles per tested gene and control gene (housekeeping gene, Gapdh) compared to negative control samples by the StepOne Plus machine (ThermoFisher, 4376600).

### Statistical analysis

We performed statistics with Prism software 9.3.1 or with R (version 4.0.5) (43). Most of the data were log_2_-transformed unless it is not written. Normal distribution was tested after this transformation. We used an unpaired t-test or Welch's test for pairwise comparisons. We analyzed multiple-group comparisons using one-way ANOVA with Dunnett's correction. For statistical analysis for proteomics see the specific section above. We used a repeated-measure two-way ANOVA test for helicase activity assay (with Tukey's correction) and for the analysis of live-imaging experiments. For the latter, we tested the normal distribution of the residuals of the data (by histograms) and used the Levene test to compare variances between the treatments within the data. Statistical tests were considered significant if adjusted p-values or FDR-corrected *P*-values ≤0.05. We show data as means ± SD (or ± SEM for helicase activity assay).

### Figures’ design

We placed and organized all the figures by using Adobe Illustrator software. We generated all the graphs by Prism or R (version 4.0.5) (43) software. We generated the graphical abstract and Figures 4A and 5G by BioRender.com.

## RESULTS

### BLM is recruited to stress granules under a variety of stress conditions

BLM is often thought of as a nuclear DNA helicase (29,30,46). However, in a recent study, we characterized the composition of SGs as a result of sodium arsenate stress and found that BLM localizes in SGs (32). Since SG composition varies as a function of the stress type (47–49), we decided to test whether BLM is present in SGs as a result of other stressors; heat shock, puromycin, and thapsigargin.

**Figure 1.**
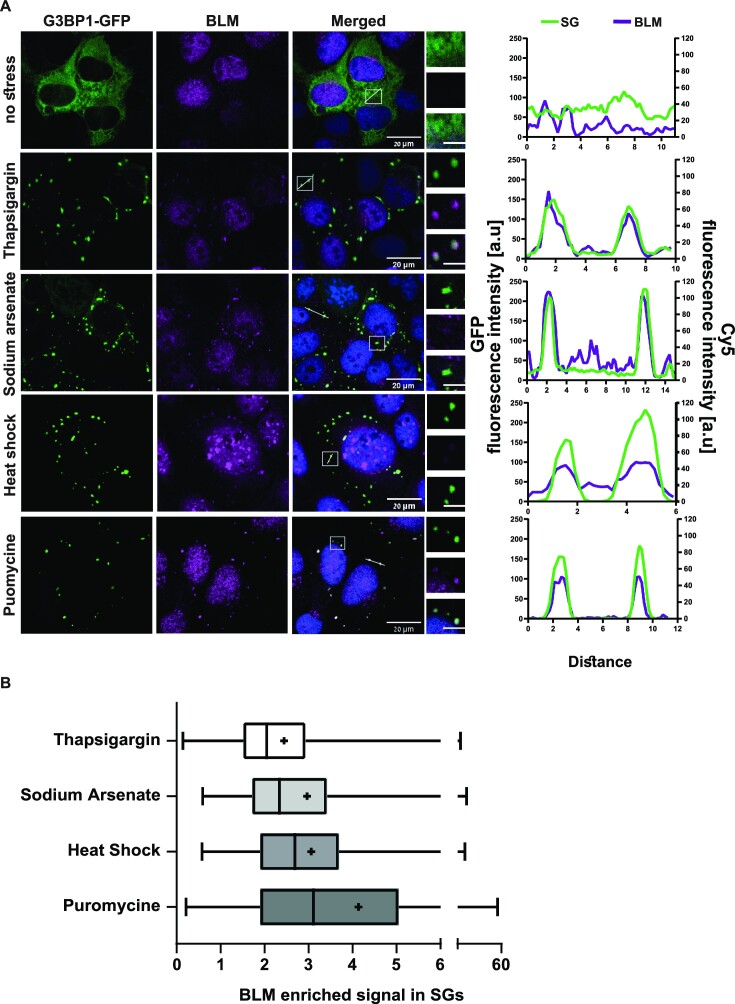
BLM is a resident protein of stress granules. (**A**) Confocal micrographs of BLM immunostaining (Cy5, Purple), in U2OS cells under a variety of stressors and co-localization with stress granule marker G3BP1-GFP (green). Nuclei (DAPI, blue). ×63 lens. Scale bar – 20 um. Inset scale bar 2 um. Intensity profiles for SGs and BLM channels in representative SGs under different stress conditions using Fiji software. (**B**) Box plot of BLM enrichment in SGs, which were quantified from micrographs of >30 cells per treatment. Median (|) and mean (+). Analyzed using Fiji software.

Under basal growth conditions, without stress, BLM was mainly located in the nucleus. However, BLM was detected within cytoplasmic G3BP1-GFP expressing SGs under all the stress conditions tested in U2OS cells (Figure 1A). The enrichment of BLM in SGs, relative to the surrounding cytoplasm, was in the range of ∼2- to 4-fold (average ×2.4 (thapsigargin), ×2.9 (sodium arsenate), ×3.0 (heat shock) and ×4.2 (puromycin), Figure 1B and Supplementary Data 1, Datasheet S2). In addition, colocalization between G3BP1-stained SGs and BLM was observed in fibroblasts and iPSC-derived neurons, under sodium arsenate stress (400 uM, 30 min, Supplementary Figure 1). This indicates that BLM is recruited to SGs, under broad types of cellular stress and in different cell lines.

### BLM directly interacts with rG4s

**Figure 2.**
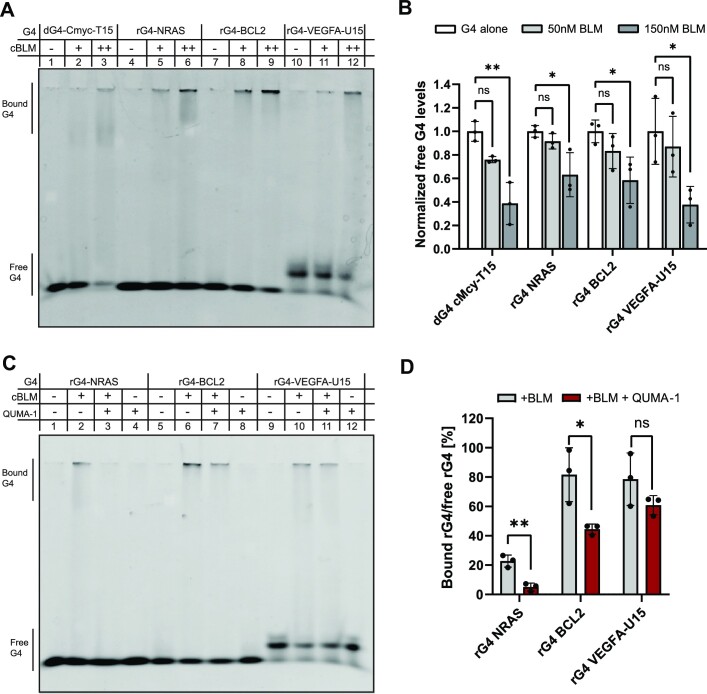
BLM binds rG4s *in vitro*. (**A**) Representative blot from an electromobility shift assay of DNA (CMyc-dG4-T15) or RNA (rG4-NRAS, rG4-BCL2 and rG4-VEGFA-U15) G4 forming sequences without/with recombinant core BLM protein (+ for 50 nM or ++ for 150 nM). (**B**) Quantification of the free rG4 signal for each of the oligos tested in panel (A). Data normalized to free G4 signal obtained from the lane of G4 without protein for each oligo (lanes 1, 4, 7, 10). Three experimental repeats. (**C**) Representative EMSA of RNA G4 forming sequences, bound to 150nM recombinant core BLM protein with/without rG4-binder QUMA-1 (1 uM). (**D**) Quantification of the normalized core BLM-bound rG4 signal with/without competition on rG4 by QUMA-1 (1 uM) for each of the oligos tested in panel **(C)**. Data normalized to the free rG4 signal obtained from the lane of rG4 without protein/QUMA-1 for each oligo (lanes 1, 5, 9). Three experimental repeats. One-way ANOVA with Dunett's test (B) and two-tailed unpaired *t*-test (D); ns, non-significant; * *P*-value < 0.05, ** *P*-value < 0.01, *** *P*-value < 0.001, **** *P*-value < 0.0001.

Since SGs are composed of RNAs and RNA-binding proteins, we hypothesized that BLM binds rG4s. We thus performed electromobility shift assays (EMSA) with a recombinant, truncated, BLM variant (amino acids 636–1298; named cBLM), which retains the translocation and unwinding activities of the full-length BLM (50–52). A series of synthetic G4-forming oligonucleotides, whose ability to adopt a G4 structure was validated by circular dichroism (CD) assays (6) (Supplementary Figure 2), was introduced in a solution containing cBLM (Figure 2 and Supplementary Figure 3A). The EMSA analysis showed that cBLM binds to rG4-NRAS (6,25), rG4-BCL2 (6,27) and rG4-VEGFA (4,53), naked or containing also a 3′ tail of U15 (rG4-VEGFA-U15, Figure 2A). In addition, cBLM bound dG4 with a 3′ tail of T15 (dG4-cMyc-T15 (27,54)), which was used as a positive control (Figure 2A, Supplementary Data 1, Datasheet S1). The EMSA quantification demonstrates that cBLM binds to both dG4s and rG4s in a concentration-dependent manner (Figure 2B and Supplementary Data 1, Datasheet S3).

For validation, in another EMSA experiment, the bands that correspond to the rG4s (dG4-cMyc-T15 or rG4-VEGFA-U15) bound to cBLM were extracted and analyzed by mass spectrometry, to reveal the nature of the predominant proteins in these bands (Supplementary Figure 3B, C). Gel areas without cBLM in lanes with rG4s alone served as negative controls, and cBLM alone served as a positive control. The main identified protein was the human BLM with peptide spectrum match score (#PSM) of ∼3–3000 times higher abundance than common laboratory contaminants and ∼100 times higher than negligible *E.coli* peptides (Thioredoxin-1, Supplementary Data 1, Datasheets S4).

To substantiate that BLM is an rG4-binding protein, we next tested whether the binding of cBLM to rG4s is affected by the presence of the specific small molecule rG4-ligand QUMA-1 (55), which has been shown to compete with rG4-protein interactions (6,56). We found that QUMA-1 reduced cBLM binding to rG4s (Figure 2C, D and Supplementary Data 1, Datasheet S5). The bound/free rG4 ratio (binding percentage) varied between different rG4-forming sequences, with the lowest binding preference of cBLM for rG4-NRAS. The competition of QUMA-1 decreased the binding capacity of cBLM to rG4-NRAS and rG4-BCL2 by approximately 50% (*P*-value = 0.0034 or 0.0262, unpaired *t*-test, respectively), while it barely affected cBLM binding to rG4-VEGFA-U15 (Figure 2D). This might be related to cBLM’s interactions with both VEGFA-U15 rG4 structure and its 3′ single-stranded uridine sequence tail (U15), which is unlikely to bind QUMA-1. Together, this series of results confirm that BLM is both an dG4 and rG4 binding protein.

### BLM unwinds rG4s *in vitro* and in cells and affects the enrichment of rG4s in SGs

**Figure 3.**
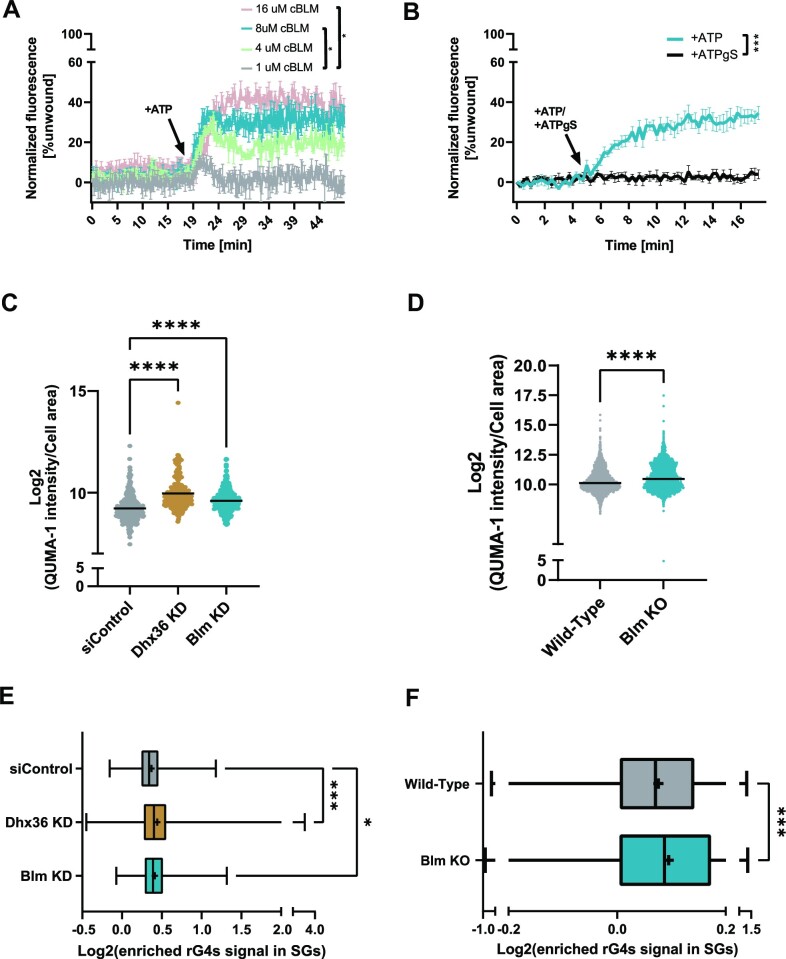
BLM unwinds rG4s *in vitro* and in cultured cells. In-vitro unwinding assay monitored in real-time, as the relative emission of a 6FAM-labelled fluorescent short oligo (% unwound) unwound from a dabcyl labelled quencher oligo, VEGFA-rG4-U15, and in response to (**A**) cBLM concentrations (1, 4, 8, 16 uM) with ATP addition (+ATP) or (**B**) 8 uM cBLM in addition of ATP or a non hydrolysable analogue ATPgS (+ATP/+ATPgS). Data normalized to 0 $\mu {\mathrm{M}}$ cBLM and to the first time point, per condition. Average of two or five technical repeats in A or B, respectively; Two-way ANOVA repeated measure with Tukey's test for multiple comparisons. Representative scatter plots of the endogenous rG4 signal gained by quantification of QUMA-1 staining in (**C**) U2OS cells with siRNA knock-down of Dhx36 or Blm. Stress induced by sodium arsenate, 150 uM, 2.5 hr. siControl non-targeting oligonucleotides served as controls, or (**D**) RPE wild-type versus BLM KO cells. Three experimental repeats; log_2_-transformed rG4 intensity in individual cell, normalized to the cell area. Horizontal line - mean. Representative box plots of endogenous rG4 signal, gained by QUMA-1 quantification in stress granules, relative to adjacent cytoplasm of (**E**) U2OS cells or (**F**) RPE wild-type versus BLM KO cells, under conditions identical to those in panels C and D. Three experimental repeats; Median (|) and mean (+) of log_2_-transformation of rG4 enriched signal in stress granules. One-way ANOVA with Dunnett's test (C, E), Two-tailed (D) or one-tailed (F) unpaired *t*-test. * *P*-value < 0.05, ** *P*-value < 0.01, *** *P*-value < 0.001, **** *P*-value < 0.0001.

Based on the above results, we examined the potential helicase activity of BLM on rG4s by an *in vitro* fluorescence-based unwinding assay (35). In this assay, a dabcyl quencher containing-rG4 oligonucleotide (S-rG4-VEGFA-U15) is hybridized with a 3′ 6FAM labelled short complementary oligonucleotide that has the potential to emit fluorescence only when unpaired from the quencher. cBLM led to the unwinding of the rG4-containing quencher, once ATP is added, triggering an increase of the FAM fluorescence. This fluorescence increase was found to be dependent on the cBLM concentration, reaching up to 40% of the unfolded rG4 at high cBLM concentrations (up to 16 uM, adjusted *P*-value = 0.0231, Two-way ANOVA repeated measure with Tukey's test; Figure 3A and Supplementary Data1, Datasheet S6).

To demonstrate that BLM functions as an ATP-dependent rG4 helicase, as previously reported for its G4 DNA helicase activity (29,57), we performed the unwinding assay with either ATP or non-hydrolyzable ATP analogue, ATPgammaS (ATPgS). We monitored a significant increase in unwinding cBLM activity in the presence of ATP as compared to ATPgS (Figure 3B and Supplementary Data 1, Datasheet S6). Therefore, BLM is an ATP-dependent rG4 helicase protein in addition to its known function as a dG4 helicase.

Next, we tested whether BLM serves as a helicase for endogenous rG4s. For this purpose, we knocked-down BLM in U2OS cells, using short interfering RNAs (siRNAs). Forty-eight hours after transfection, the levels of BLM were decreased by approximately 50% (Supplementary Figure 4 and Supplementary Data 1, Datasheets S7 and S8). We then added QUMA-1 (0.5 uM, 3 hr). ImageStream analysis revealed higher levels of endogenous rG4 after BLM knock-down (Figure 3C and Supplementary Data 1, Datasheet S9) and similar results were obtained after knocking-down DHX36 expression, a known rG4 helicase (58). Orthogonally, ImageStream analysis of QUMA-1 intensities demonstrated that BLM KO RPE cells display a higher QUMA-1 fluorescence, attributed to rG4 *bona fide* signal, as compared to wild-type RPE cells (Figure 3D and Supplementary Data 1, Datasheet S10). This indicates that BLM indeed acts as an rG4 helicase in cells.

In addition, because rG4s accumulate and regulate SG formation (6,15,17,59), the knock-down of either BLM or DHX36 in U2OS cells resulted in a higher levels of SG-associated rG4s, as compared to control (Figure 3E and Supplementary Data 1, Datasheet S11), supporting that BLM regulates rG4s levels also within SGs.

Finally, we quantified the SG-enriched rG4 signal as a result of BLM KO in RPE cells (Figure 3F). G3BP1-stained SGs contained a higher rG4 signal in the BLM KO RPE cells compared to wild-type RPE cells (Figure 3F and Supplementary Data 1, Datasheet S12). We note that the enrichment of the rG4 signal within SGs in RPE cells was generally lower than in the U2OS cells. Collectively, this series of results confirmed that BLM regulates rG4 levels in SGs.

### BLM is recruited to SGs through rG4 binding under stress

rG4 interactions contribute to SG formation (6). In this context, we further asked how rG4s recruit BLM to SGs. Under sodium arsenate-induced stress, we used APEX proximity labelling (60–62) to explore the impact of rG4 availability on the SG proteome. We induced G3BP1-APEX or nuclear export signal-APEX (NES-APEX) expression in U2OS cells (for labelling the SG proteome or cytoplasmic background, respectively) that we previously developed ((32), and Supplementary Figure 5). Cells were incubated with QUMA-1 (1 uM, 3 hr) to sequester rG4s or with DMSO (carrier; as control) and subjected to stress (150 uM sodium arsenate, 2.5 hr). APEX activity was induced with biotin phenol and hydrogen peroxide for biotin labelling of SG proteins. By western blot (WB) analysis, we confirmed the comparable biotinylated protein pattern for each of the APEX baits (NES or G3BP1) either with QUMA-1 or DMSO (Supplementary Figure 6). Furthermore, when the APEX labelling was not activated, the background levels of detected proteins were negligible (Supplementary Figures 5 and 6). Therefore, QUMA-1 does not affect APEX labelling directly.

**Figure 4.**
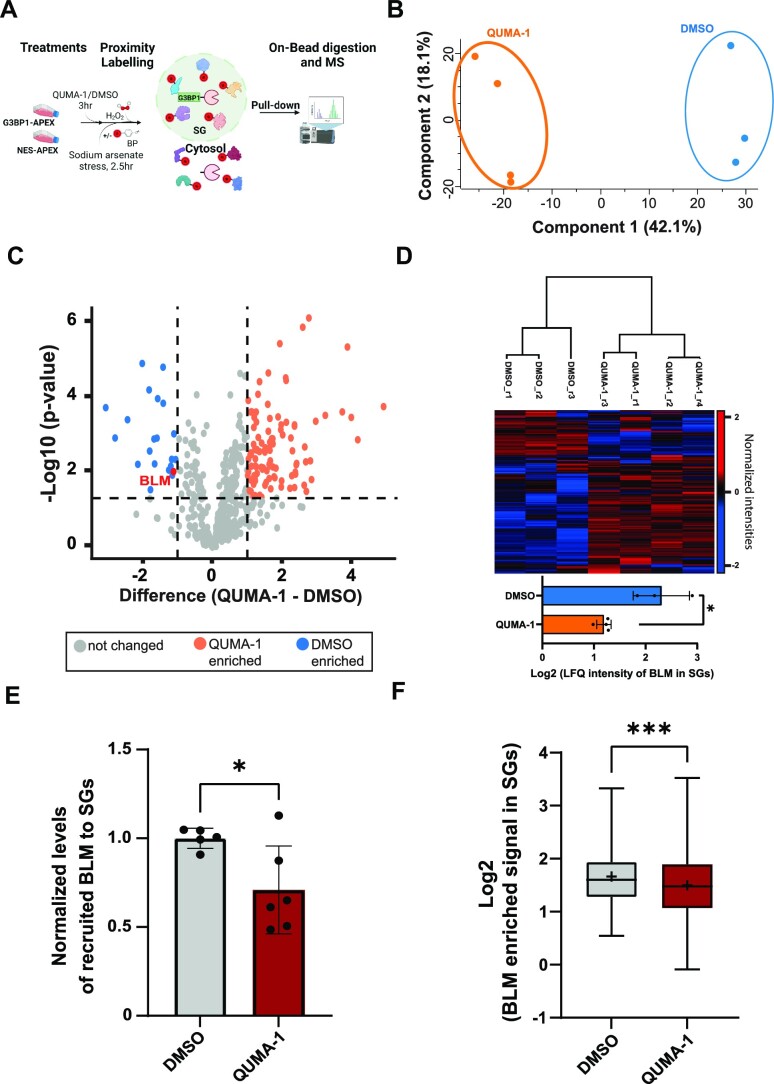
BLM is recruited to SGs in an rG4-dependent manner. (**A**) Diagram of the experimental design. (**B**) Principal component analysis of the proteomic content of APEX-isolated stress granules under QUMA-1 treatment (1 uM, 3 hr) or control (carrier, DMSO). (**C**) A volcano plot of APEX-isolated SG proteins, obtained under QUMA-1 treatment (orange), relative to DMSO control (blue). Y-axis –log_10_ of *P*-value (*P*-value < 0.05) and x-axis, log_2_ values of fold-change. BLM is highlighted in red. Grey proteins were lacking statistical significance. (**D**) Heatmap of unsupervised clustering of the final 472 SG-associated proteins that were enriched or depleted under experimental conditions in stress granules (upper), and bar plot represents BLM’s intensity under QUMA-1 or DMSO conditions (orange/blue, lower). FDR corrected *P*-value (* adjusted *P*-value < 0.05). (**E**) APEX-isolated SG proteins blotted with anti-BLM antibody after QUMA-1 treatment normalized to DMSO control. BLM is represented at ∼169 kDa. (**F**) Representative box plot of BLM enrichment in SGs. Quantification of immuno-stained confocal micrographs with median (|) and mean (+) of log_2_-transformation of BLM enriched signal in SGs, relative to adjacent cytoplasm of U2OS cells. Three experimental repeats. Two-tailed unpaired *t*-test, * *P*-value < 0.05 (E),*** *P*-value < 0.001 (F).

Following the pull-down of biotinylated proteins, shotgun mass spectrometry identified proteins that are enriched or depleted in SGs, in response to the availability of cellular rG4s (Figure 4A and Supplementary Figure 7). Overall, 472 SG-associated proteins were identified with good confidence (adjusted *P*-value < 0.05, FDR correction, log_2_FC > 0). Of these, 90 proteins were enriched and 20 depleted when rG4s were sequestered by QUMA-1 (adjusted *P*-value < 0.05, FDR correction, log_2_FC > 1). Clustering of experimental repeats between the proteomes affected by QUMA-1 *versus* control treatment was satisfying (Figure 4B) and the differences in proteome composition in response to sequestration of rG4s by QUMA-1 were quantified (Figure 4C, D and Supplementary Data 1, Datasheets S13 and S14).

BLM was relatively depleted from SG proteome in cells treated with QUMA-1, suggesting that rG4 levels and/or their accessibility affect its recruitment (adjusted p-value = 0.03; Figure 4C, D). This observation was orthogonally validated by a WB analysis of BLM, which was pulled down after G3BP1-APEX labelling under stress conditions (Figure 4E and Supplementary Data 1, Datasheet S15). In addition, a reduction in BLM abundance in SGs was observed by fluorescence microscopy in stained cells that were treated with QUMA-1 or DMSO (carrier; as control) prior to their fixation (Figure 4F and Supplementary Data 1, Datasheet S16). In conclusion, BLM is a SG-associated rG4 helicase that is recruited to SGs in an rG4-dependent manner.

### BLM inhibits SG formation

**Figure 5.**
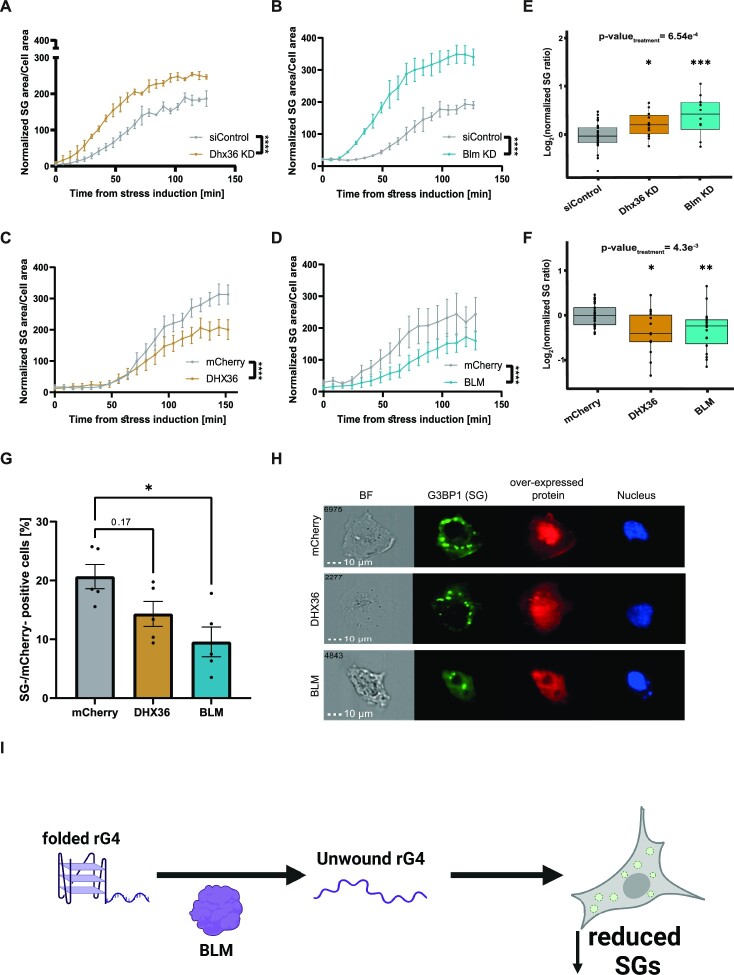
BLM negatively regulates SG formation. Quantification of the ratio of SG area, to the cell area, by live imaging of G3BP1-GFP in U2OS cells treated with 150 uM of sodium arsenate for 2.5 hr. siRNA knockdown of (**A**) Dhx36, or (**B**) Blm compared to siControl. Over-expression of (**C**) mCherry-DHX36, or (**D**) mCherry-BLM compared to mCherry-only overexpression control. Four sites per well, 3–4 wells per condition. Three independent repeats. Two-way ANOVA repeated measures with FDR correction, **** *P*-value < 0.0001. (**E**) Box plot quantification of data from (A, B) and (**F**) Box plot quantification of data from (C, D), 120 min after stress induction. Data normalized to control average, per repeat. One-way ANOVA with Dunnett's test, *P-value < 0.05, **P-value < 0.01, ***P-value < 0.001. (**G**) Bar plot of the percentage of stress granule-positive U2OS cells with overexpression of mCherry-DHX36 or mCherry-BLM, compared mCherry only as a control. ImageStream study, one-way ANOVA with Dunett's test, *P-value < 0.05. (**H**) Representative micrograph channels: G3BP1-GFP (green), mCherry (red), Hoechst 44432 (Blue). (**I**) A model for the regulatory role of BLM in SG formation through the unwinding of rG4. BLM is recruited to SGs, where plausibly it performs its rG4 helicase activity.

This wealth of data led us to postulate that BLM may regulate SG formation, as rG4s affect SG formation (6), recruit BLM to SGs (Figure 4) and BLM likely unwinds rG4s (as demonstrated *in vitro* and in cells, Figure 3). To investigate this, we manipulated BLM levels in U2OS cells. Live-cell imaging revealed that SG formation was increased by knocking down BLM or DHX36 levels, relative to control (Figure 5A, B, E and Supplementary Data 1, Datasheet S17). Accordingly, the overexpression of mCherry-BLM or mCherry-DHX36 inhibited SG formation, relative to mCherry overexpression control (Figure 5C, D, F and Supplementary Data 1, Datasheet S18). eIF2-alpha phosphorylation was not induced by the overexpression of mCherry, suggesting that it did not affect the cellular stress response by itself (Supplementary Figure 8, and Supplementary Data 1, Datasheet S19).

Orthogonally, we detected lower percentages of cells that harbor visible SG in cultures that express mCherry-BLM or mCherry-DHX36, relative to cultures that expressed mCherry alone (Figure 5G, H and Supplementary Data 1, Datasheet S20). Altogether, we suggest a model whereby BLM is a SG-associated rG4 helicase that negatively regulates SG formation *via* unwinding of rG4s (Figure 5I).

## DISCUSSION

In this study, we demonstrate that BLM, a known nuclear DNA G4 helicase, also localizes to cytosolic SGs, under a variety of stress conditions and in different cell types. In SGs, BLM binds to and unwinds endogenous RNA G4s. Furthermore, BLM was found to be recruited into SGs in an rG4-dependent manner and regulates their formation.

We propose that cellular BLM levels alter SG formation *via* rG4 unwinding, which is in line with our recent observation that rG4-protein interactions and rG4 availability contribute to SG formation (6). This hypothesis is further substantiated by both the results collected thanks to the newly developed *in vitro* cBLM helicase activity and previous cellular results showing that rG4 accumulation in SGs is prevented by DHX36, which then leads to SG reduction (17).

Nuclear breakdown as a result of oxidative stress, leads to the presence of fragmented DNA in the cytoplasm. Some of these DNA fragments form dG4s that accumulate within SGs (63). In this context, it is intriguing that BLM binds to rG4s and is incorporated into SGs under a broad variety of stress conditions, some of which do not inflict nuclear breakdown. However, our model does not rule out the possibility dG4s might regulate SG biology independently of rG4s.

rG4s were suggested to trigger and/or maintain RNA-driven phase separation (15,64). As an example, the transfection of G4C2 rG4-forming sequences elicits robust SG assembly (15), and consistently, rG4 stabilization is enhanced under stress in cells (12). DMS-seq analysis showed that stress induces widespread folding of mRNAs into rG4s, raising the possibility that they may regulate mRNA metabolism in a stress-dependent manner. Moreover, rG4s are also involved in the regulation of paraspeckles (65), suggesting a broader function in condensates. The number of G-quartets certainly affects the propensity to de-mix (15,64) and we have recently demonstrated that the rG4-specific binder QUMA-1 competes with G3BP1 for binding to rG4s *in vitro*, and inhibits SG formation (6). As rG4s plausibly contribute to biomolecular condensate valency, perhaps sequestration of rG4s by QUMA-1 inhibits overall SG formation by the reduction in valency.

It is important to better understand the mechanisms by which BLM regulates SG formation, as well as, the mechanisms underlying BLM recruitment to SGs, in addition to rG4 interaction. Intriguingly, BLM protein undergoes phase separation *in vitro* (66), supporting its potential involvement in biomolecular condensates. In this context, SUMOylated BLM has been reported to be localized to PML nuclear bodies (67), and we have shown that SUMOylation controls G3BP1 in stress granules (32). Therefore, it might be that SUMO or other post-translational modifications also regulate BLM’s localization to SGs. If helicases such as BLM or DHX36 (17) unwind SG-enriched rG4s, they might reduce rG4 availability to bind to G3BP1 (6,25). To date, the roles of rG4 binding proteins in phase separation are not well understood. However, an emerging hypothesis is that rG4 helicases could control facets of the RNA-protein network that govern condensate dynamics.

In summary, our study reveals a new role for BLM in RNA biology in general, and under stress conditions in particular, which likely occurs *via* the targeting and unwinding of endogenous rG4s. The study emphasizes the crucial regulatory role of rG4s and related helicases in SG dynamics and suggests that additional explorations of RNA-associated BLM functions are warranted in the context of G4-associated genetic diseases and specifically in Bloom's syndrome.

## Supplementary Material

gkad613_Supplemental_Files

## Data Availability

Further information and requests for resources/reagents should be directed to and will be fulfilled by the Lead Contact, Prof. Eran Hornstein (eran.hornstein@weizmann.ac.il). All plasmids are availble via addgene.org. The mass spectrometry proteomics data (Figure 4 and Supplementary Figure 3) have been deposited to the ProteomeXchange Consortium via the PRIDE (68) partner repository with the dataset identifiers: PXD042983 (gel-based MS experiment) and PXD039534 (APEX proximity labelling proteomics experiment).
