# Supplementary material for: BLM helicase protein negatively regulates stress granule formation through unwinding RNA G-quadruplex structures
Source: Nucleic Acids Res. 2023 Jul 28;51(17):9369–84. doi: 10.1093/nar/gkad613 (PMC10516661; doi:10.1093/nar/gkad613)
Supplement: gkad613_Supplemental_Files [file gkad613_supplemental_files.zip › Danino et al_Supplementary_RevisedFigures_200623.pdf]

Supplementary Information

BLM helicase protein negatively regulates stress granule formation through unwinding RNA G-quadruplex structures

Yehuda M. Danino[1,2], Lena M. Molitor[1,2], Tamar Rosenbaum-Cohen[2,3], Sebastian Kaiser[4], Yahel Cohen[1,2],, Ziv Porat[5], Hagai Marmor-Kollet[1,6], Corine Katina[7], Alon Savidor[7], Ron Rotkopf[8], Eyal Ben-Isaac[9], Ofra Golani[9], Yishai Levin[7], Ian D. Hickson[4], David Monchaud[10] and Eran Hornstein[1,2]*

Affiliations

[1] Department of Molecular Genetics, Weizmann Institute of Science, Rehovot 7610001, Israel.
[2] Department of Molecular Neuroscience, Weizmann Institute of Science, Rehovot 7610001, Israel.
[3] Department of Brain science, Weizmann Institute of Science, Rehovot 7610001, Israel.
[4] Center for Chromosome Stability, Dept. of Cellular and Molecular Medicine, Copenhagen Univ, 2200 København N., Denmark.
[5] Flow Cytometry Unit, Life Sciences Core Facilities, Weizmann Institute of Science, Rehovot 7610001, Israel.
[6] Current address: 1E therapeutics, Rehovot, Israel.
[7] de Botton Institute for Protein Profiling, The Nancy and Stephen Grand Israel National Center for Personalized Medicine, Weizmann Institute of Science, Rehovot 7610001, Israel.
[8] Bioinformatics Unit, Life Sciences Core Facilities, Weizmann Institute of Science, Rehovot 7610001, Israel.
[9] MICC Cell Observatory Unit, Life Sciences Core Facilities, Weizmann Institute of Science, Rehovot 7610001, Israel.
[10] Institut de Chimie Moleculaire, ICMUB CNRS UMR 6302, UBFC Dijon, France.

*To whom correspondence should be addressed.
Eran Hornstein Eran.Hornstein@weizmann.ac.il;

Supplementary Figures

**Supplementary Figure 1**	BLM is colocalized with stress granules in non-cancer cell lines including fibroblasts and iPSC-derived neurons
**Supplementary Figure 2**	CD profiles confirm the G4 structures of the oligos that are newly used in this study
**Supplementary Figure 3**	Validation of recombinant human cBLM protein purity and binding to both DNA and RNA G4s in vitro, by mass-spectrometry analysis
**Supplementary Figure 4**	Validations of Dhx36 or Blm knockdown by siRNAs that are used in this study
**Supplementary Figure 5**	Immunofluorescence staining validation of APEX proximity labelling function
**Supplementary Figure 6**	QUMA-1 does not affect APEX proximity labelling levels compared to the DMSO condition
**Supplementary Figure 7**	Proteomics analysis pipeline, comparison of technical repeats, and background analysis in data of APEX proximity labelling proteomics experiment
**Supplementary Figure 8**	mCherry overexpression does not affect stress in cells

Supplementary Figure 1

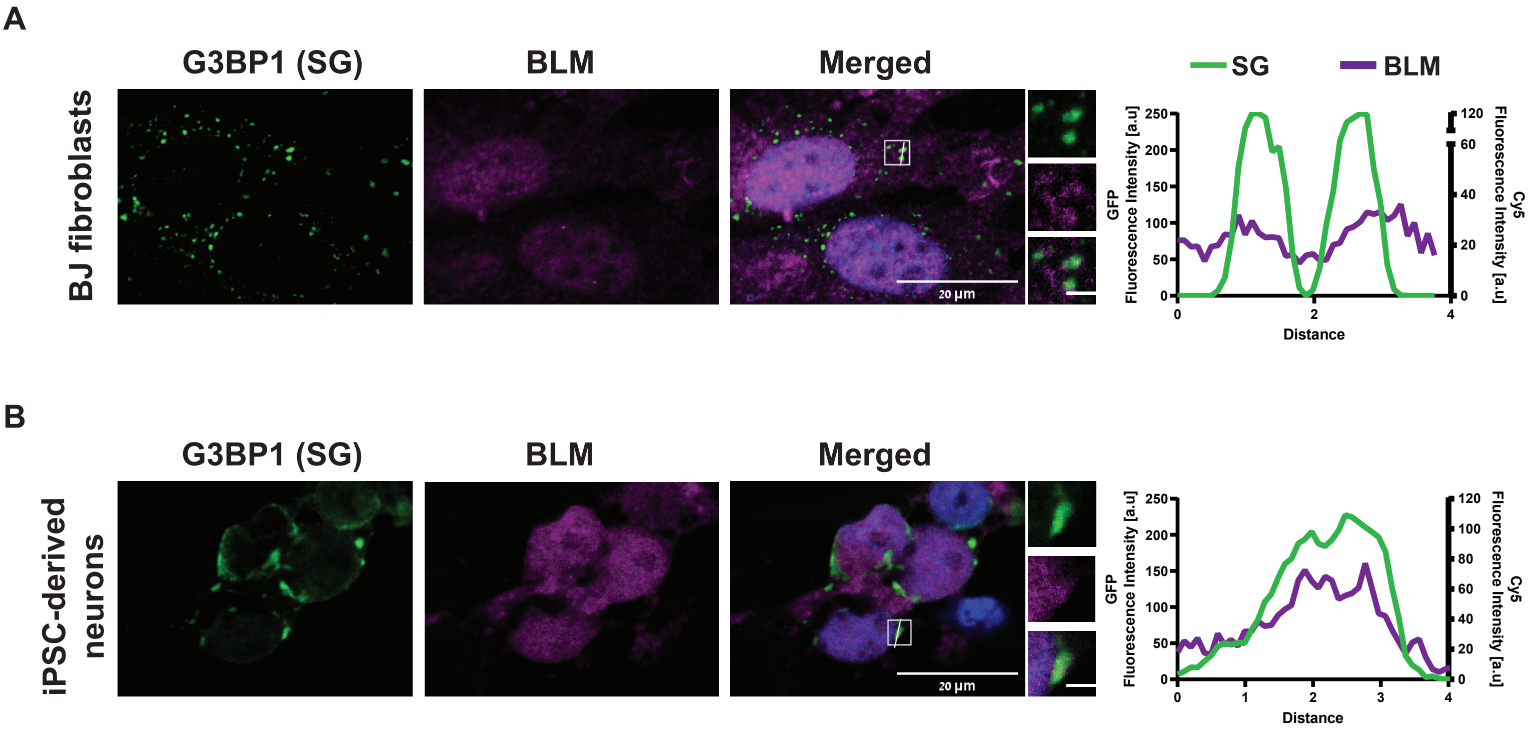

Supplementary Figure 1. BLM is colocalized with G3BP1 to stress granules in non- cancerous fibroblasts and iPSC-derived neurons. Confocal immunostaining micrographs of BLM (Cy5, Purple), stress granule marker G3BP1 (Cy2, Green) and Nuclei (DAPI, blue), in (A) BJ fibroblast cells and in (B) iPSC-derived neurons under sodium arsenate stress (400 uM, 30 min) x63 lens. Scale bar - 20 um. Inset scale bar 2 μ
representative SGs, using Fiji software.

Supplementary Figure 2

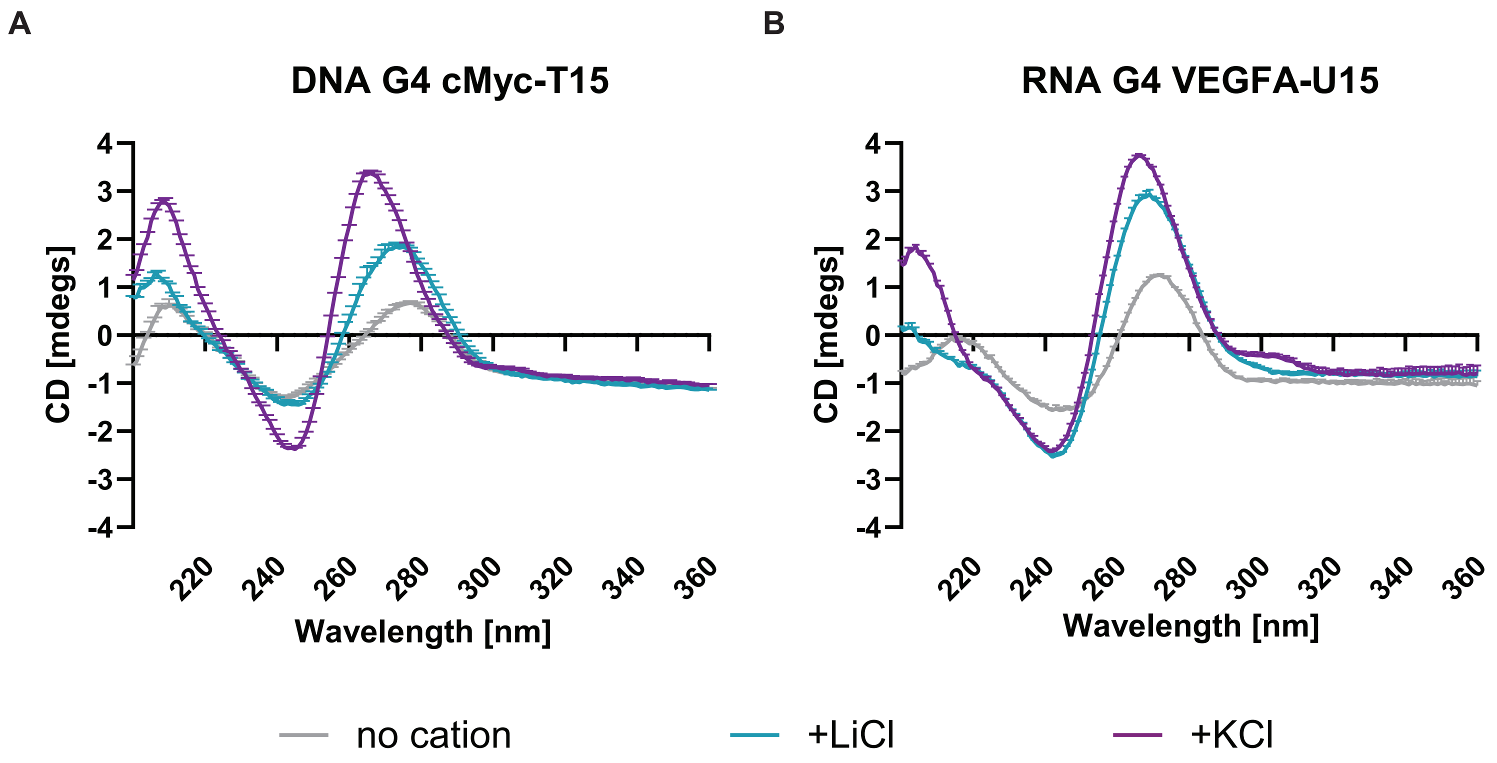

Supplementary Figure 2. CD profiles confirm the G4 structures of the oligos that are newly used in this study. CD profiles of the 6FAM-labelled (A) DNA G4-forming sequence dG4-cMyc-T15, and (B) RNA G4-forming sequence rG4-VEGFA-U15, ± access potassium/lithium ions (150 mM KCl/LiCl). The CD spectra show larger positive and negative peaks at ~ 264 and 240 nm respectively, under K+ conditions compared to Li+ or without ion conditions, suggestive of DNA or RNA G4 formation.

Supplementary Figure 3

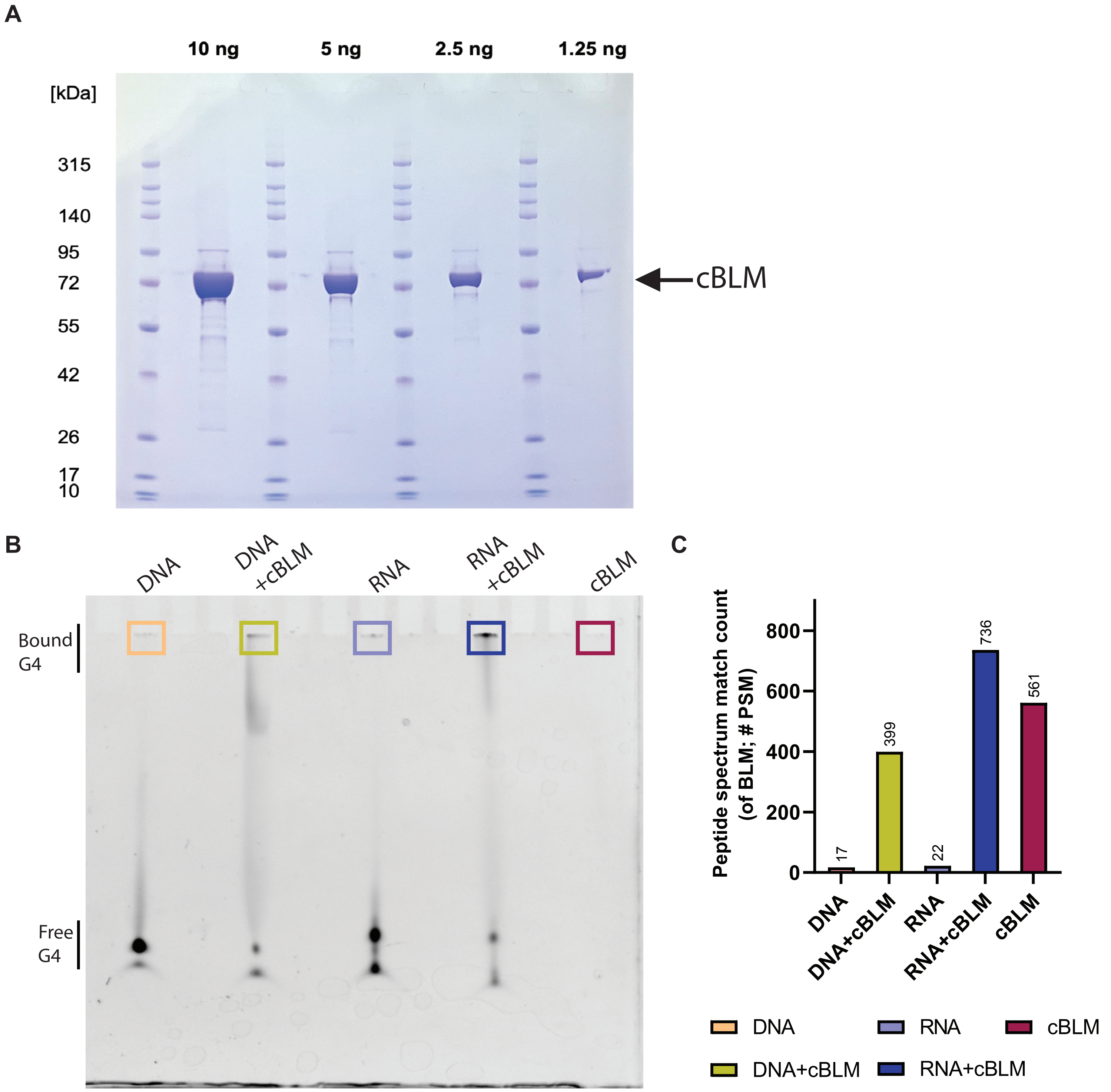

Supplementary Figure 3. Validation of recombinant human cBLM protein purity and binding to both DNA and RNA G4s in vitro, by mass-spectrometry analysis. (A) Coomassie blue staining of SDS-polyacrylamide gel loaded with different amounts of the purified human cBLM protein. (B) EMSA of FAM-labelled cMyc-A15 dG4 or VEGFA rG4, without / with 150 nM of cBLM. cBLM, without DNA/RNA, was also loaded into the gel as a control. Rectangles represent the gel bands that correspond to cBLM (bound to rG4s (dG4-cMyc-T15 or rG4-VEGFA-U15, or free)), that were extracted and analyzed by mass spectrometry, and (C) resulting semi-quantitative analysis of cBLM spectra peptide counts (peptide spectrum match; PSM), by Sequest search algorithm, to estimate the cBLM abundance in the samples.

Supplementary Figure 4

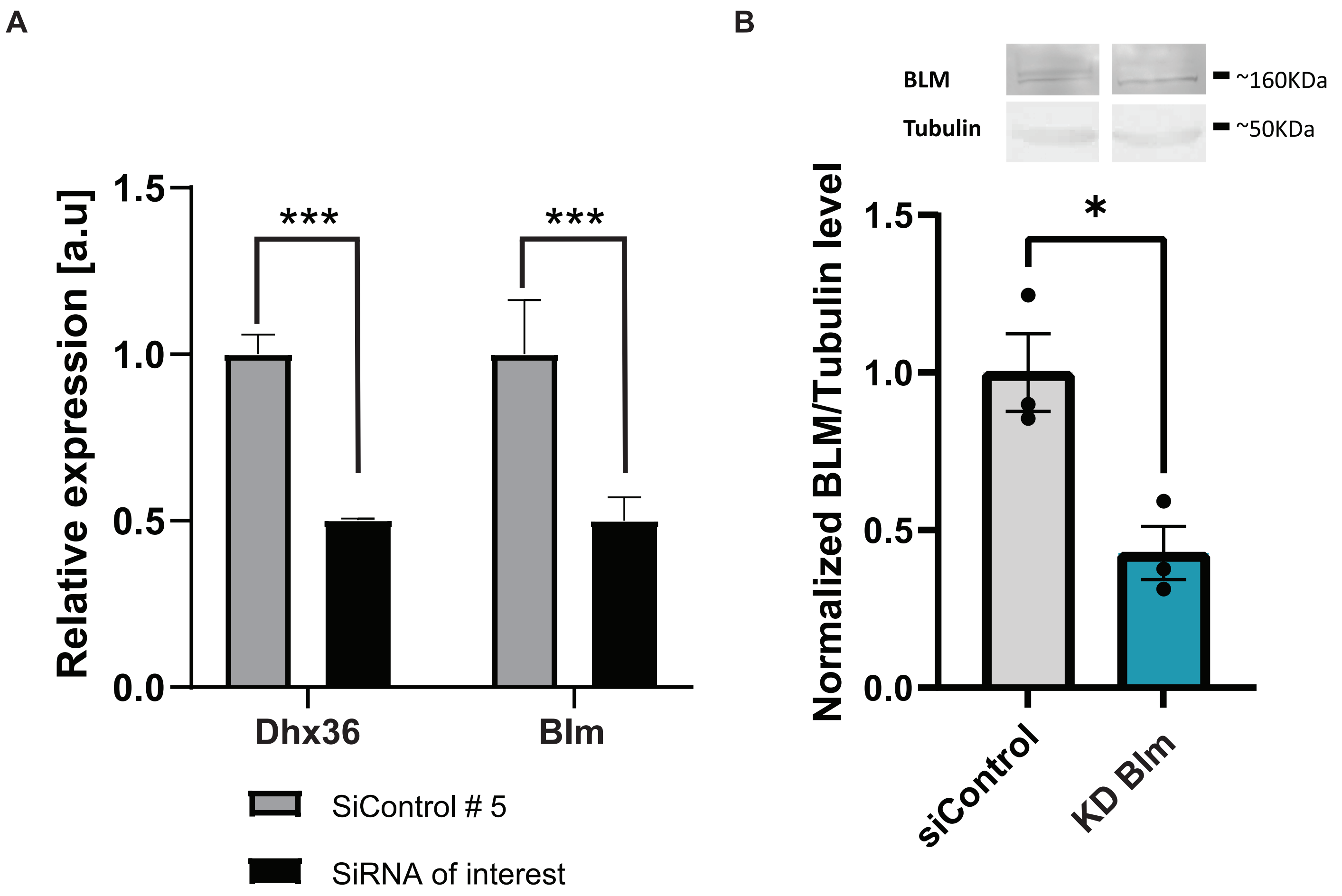

Supplementary Figure 4. Validations of Dhx36 or Blm knockdown by siRNAs that are used in this study. (A) Quantitative real-time polymerase chain reaction (rtPCR) was done to validate reduction of the expression levels of Dhx36 or Blm mRNAs as a result of siRNA treatment compared to the siControl treatment. The levels were normalized to the average of the control treatment. (B) WB analysis of BLM protein level (~160 kDa) normalized to Tubulin as control (~50 kDa) from U2OS cell lysates under siControl (non-targeting) or Blm siRNA treatment. Bradford protein assay was used to load an even amount of protein lysates and analysis was performed by Fiji software. Representative bands are presented. * p-value < 0.05, *** p-value < 0.001. Two-tailed unpaired t-test. n=3 per treatment for each of the validations.

Supplementary Figure 5

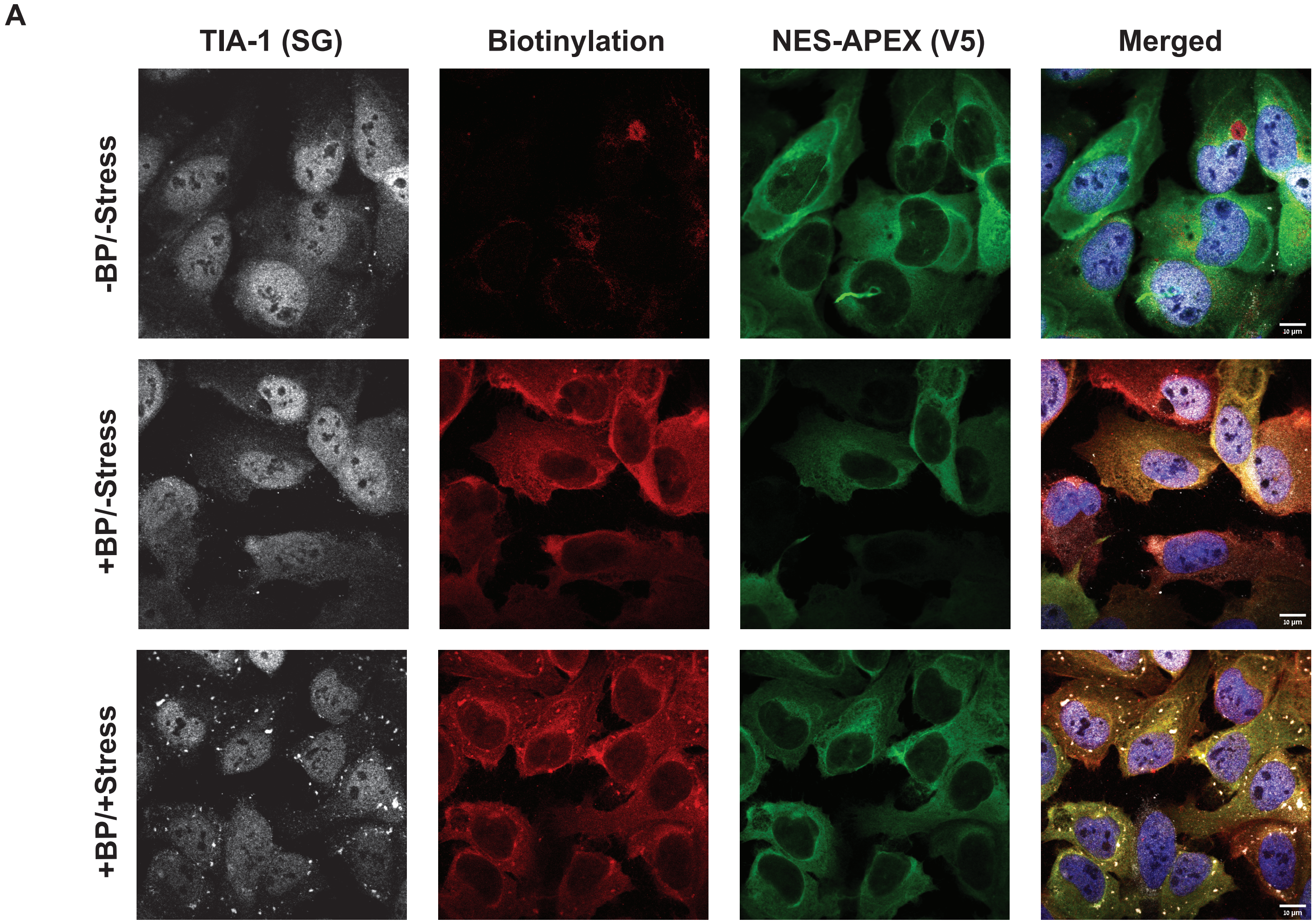

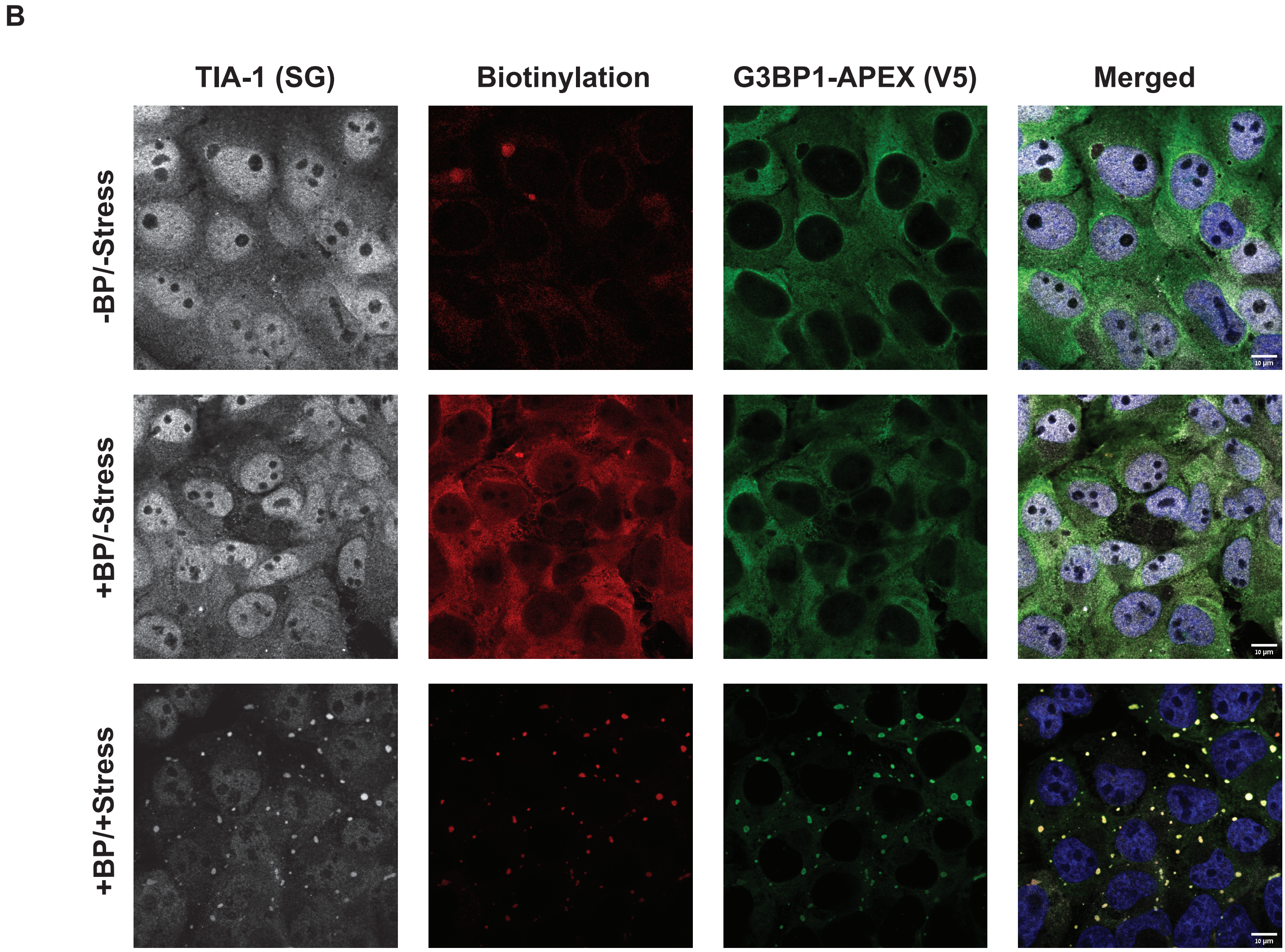

Supplementary Figure 5. Immunofluorescence staining validation of APEX proximity labelling function. Confocal micrographs representing (A) NES-APEX or (B) G3BP1-APEX biotinylation activity in U2OS cells with or without biotin-phenol (BP). Immunofluorescence depiction of TIA1 as stress granule marker (SG; grey), neutravidin-Texas-red staining of biotinylated proteins (Biotinylation; red) at the proximity of the APEX bait (V5; green), with or without sodium arsenate stress (NaAsO2 400 uM, 30 min). Merged signal demonstrating the precise localization of the APEX activity in the cytoplasm or in SGs. Lens, ×63; scale bar, 10 um

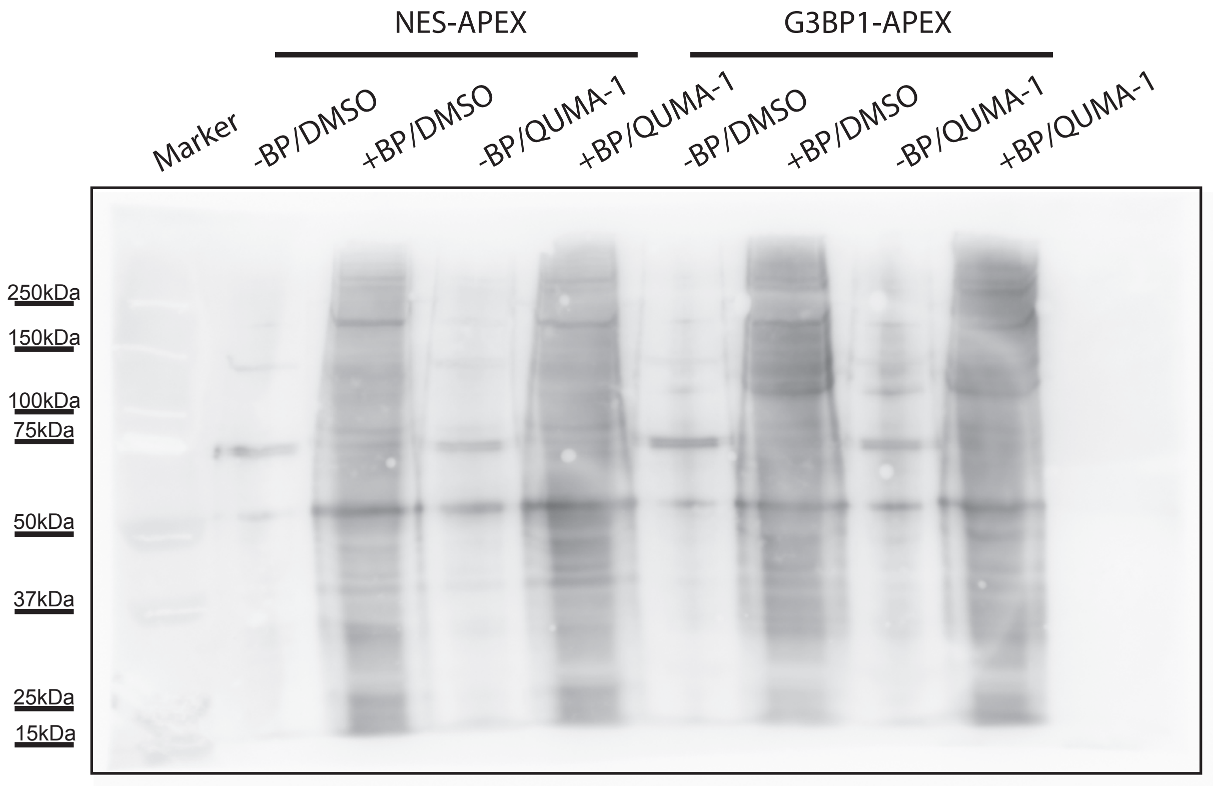

Supplementary Figure 6. QUMA-1 does not affect APEX proximity labelling levels compared to the DMSO condition. WB analysis of biotinylated proteins from whole cell lysate, reflecting NES- or G3BP1-APEX activity with or without biotin-phenol (+/-BP) under DMSO or QUMA-1 treatments. Only endogenously biotinylated proteins are observed in lanes wherein APEX was not activated (without BP; -BP).

Supplementary Figure 7

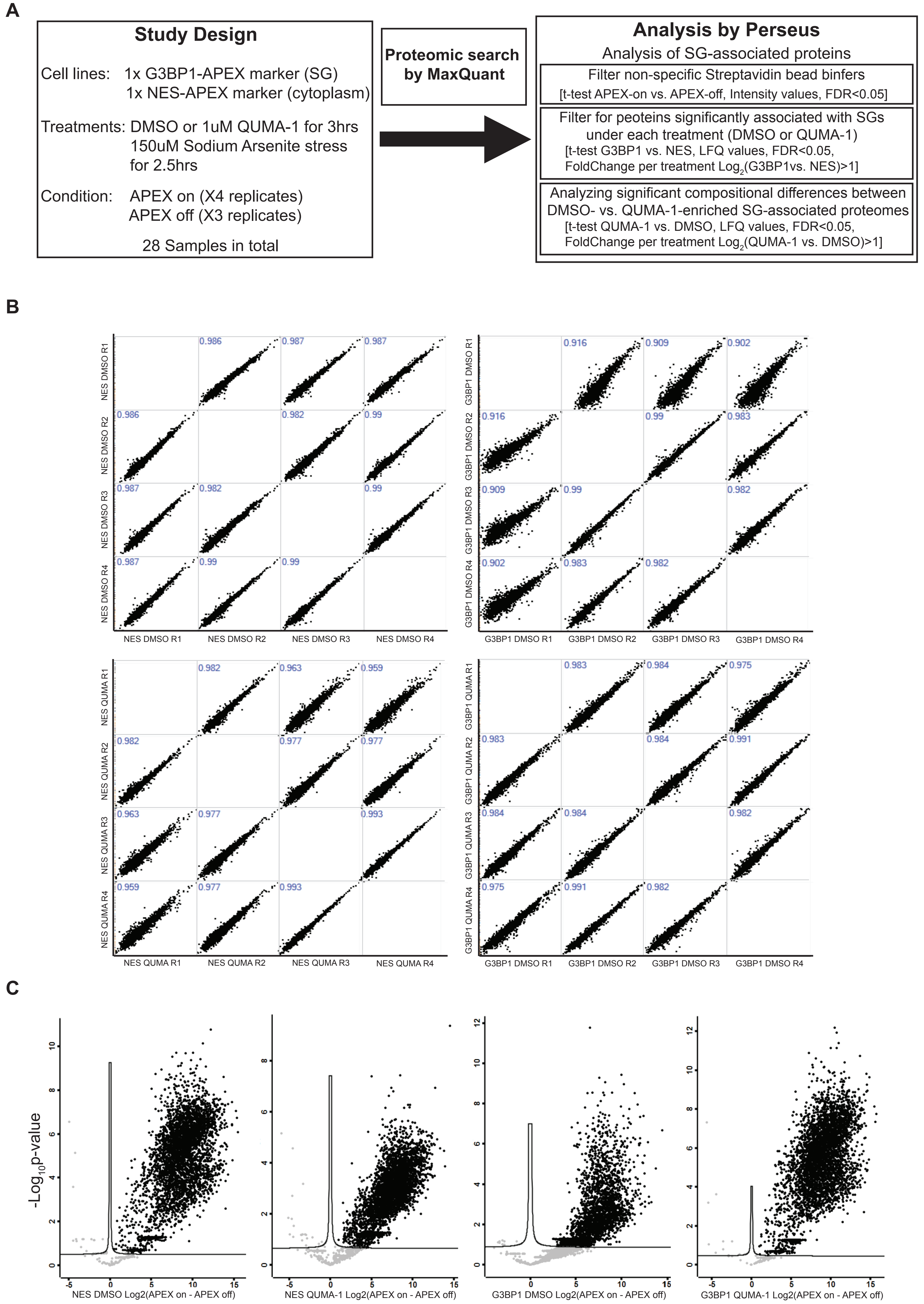

Supplementary Figure 7. Proteomics analysis pipeline, comparison of technical repeats, and background analysis in data of APEX proximity labelling proteomics experiment. (A) Analysis pipeline diagram of APEX experiment under DMSO versus QUMA-1 treatments. (B) Scatter plot and Pearson correlation coefficient values comparing technical replicates for any of the four different groups in the experiment (NES- or G3BP1-APEX markers under DMSO or QUMA-1 treatments, LFQ values). (C) Volcano plots of relative protein levels in activated APEX samples relative to non-activated APEX samples (with or without biotin-phenol; 'APEX-on' versus 'APEX-off', respectively, which is represented by the X-axis (log2 scale)). Y axis represents the differential expression p-values (−log10 scale). Proteins that are specifically bound to streptavidin beads are shown in black. Student t-test with FDR correction. adj.p-value < 0.05.

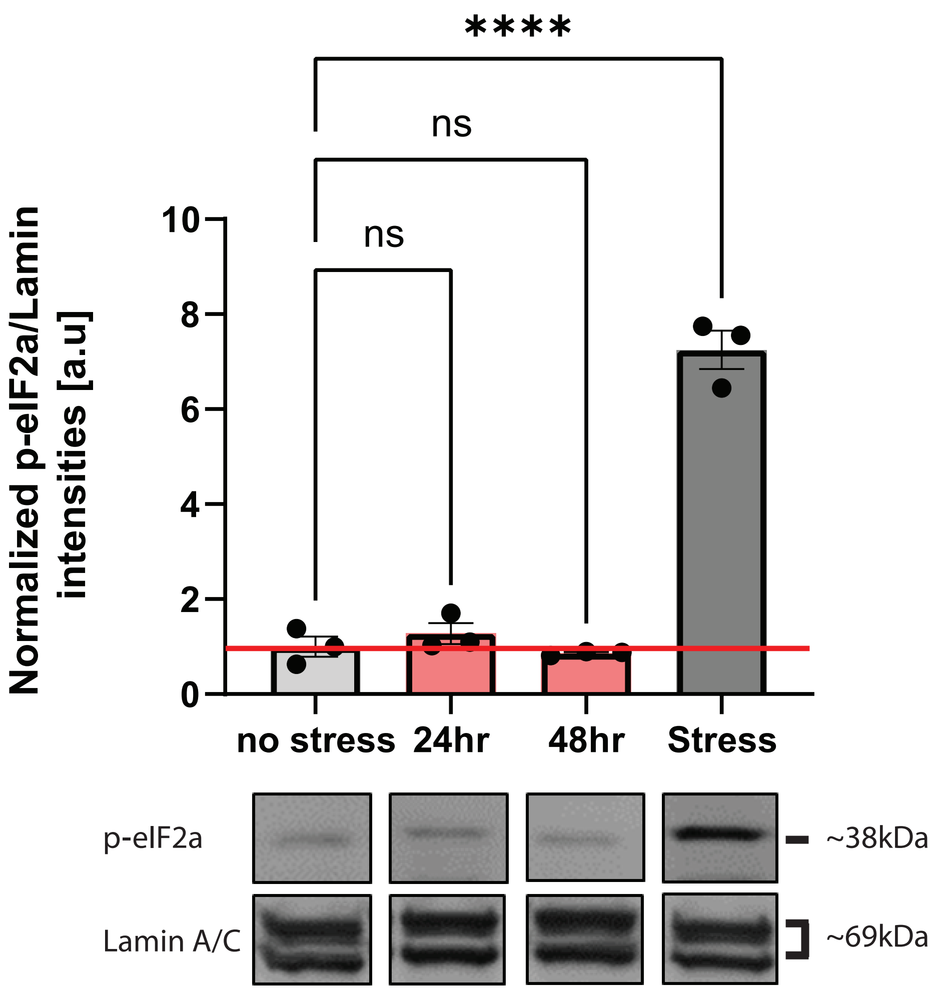

Supplementary Figure 8. mCherry overexpression does not affect stress in cells. WB analysis of expression level of p-eIF2a, a marker of unfolded protein response (~38 kDa), normalized to Lamin A/C as a control (~69 kDa) from U2OS cell lysates after 24 hr or 48 hr of transient mCherry overexpression, or under 400 uM sodium arsenate for 30 min stress without overexpression ('Stress'). The p-eIF2a/Tubulin levels were normalized to 'no stress' condition. Bradford protein assay was used to load an even amount of protein lysates and analysis was performed by Fiji software. Representative bands are presented. **** p-value < 0.0001. ns- non-significant. One-way ANOVA with a Dunett's test. Triplicates for each treatment.
